# Perfluorooctanesulfonic acid (PFOS) antagonizes gamma-aminobutyric acid (GABA) receptors in larval zebrafish and mammalian models

**DOI:** 10.1093/toxsci/kfaf101

**Published:** 2025-07-23

**Authors:** Renee Owen, Gabriel de Macedo, Jana Nerlich, Ilka Scharkin, Kristina Bartmann, Jonas Döbler, Beatrice Engelmann, Ulrike E Rolle-Kampczyk, David Leuthold, Sebastian Gutsfeld, Nicole Schweiger, Tamara Tal

**Affiliations:** Department of Ecotoxicology, Chemicals in the Environment Research Section, Helmholtz Centre for Environmental Research—UFZ, 04318 Leipzig, Germany; Department of Molecular Biology and Biochemistry, Federal University of Santa Maria, 97105-900 Santa Maria, Brazil; Medical Faculty, Carl Ludwig Institute of Physiology, University of Leipzig, 04103 Leipzig, Germany; IUF—Leibniz Research Institute for Environmental Medicine, 40225 Düsseldorf, Germany; IUF—Leibniz Research Institute for Environmental Medicine, 40225 Düsseldorf, Germany; DNTOX GmbH, 40223 Düsseldorf, Germany; Institute of Biochemistry and Biotechnology, Martin Luther University Halle-Wittenberg, 06120 Halle/Saale, Germany; Department of Molecular Toxicology, Chemicals in the Environment Research Section, Helmholtz Centre for Environmental Research—UFZ, 04318 Leipzig, Germany; Department of Molecular Toxicology, Chemicals in the Environment Research Section, Helmholtz Centre for Environmental Research—UFZ, 04318 Leipzig, Germany; Department of Ecotoxicology, Chemicals in the Environment Research Section, Helmholtz Centre for Environmental Research—UFZ, 04318 Leipzig, Germany; Department of Ecotoxicology, Chemicals in the Environment Research Section, Helmholtz Centre for Environmental Research—UFZ, 04318 Leipzig, Germany; Department of Ecotoxicology, Chemicals in the Environment Research Section, Helmholtz Centre for Environmental Research—UFZ, 04318 Leipzig, Germany; Department of Ecotoxicology, Chemicals in the Environment Research Section, Helmholtz Centre for Environmental Research—UFZ, 04318 Leipzig, Germany; Medical Faculty, University Leipzig, 04103 Leipzig, Germany

**Keywords:** zebrafish, neurotoxicology, behavior, PFOS, GABA

## Abstract

Per- and polyfluoroalkyl substances are a class of synthetic chemicals detected ubiquitously in the environment, humans, and wildlife. Perfluorooctanesulfonic acid (PFOS) is one prevalent chemical previously shown to cause adverse effects on nervous system function across in vivo and in vitro models, including dark-phase hyperactivity in larval zebrafish. The objective of this study was to evaluate the role of gamma-aminobutyric acid receptors (GABARs), GABA_A_R and GABA_B_R, as mediators of dark-phase hyperactivity in PFOS-exposed larval zebrafish. Zebrafish were acutely exposed to 7.87 to 120 μM PFOS, 0.68 to 12.4 μM picrotoxin (GABA_A_R antagonist), 0.77 to 14.05 μM propofol (GABA_A_R-positive allosteric modulator), 4.4 to 80 μM saclofen (GABA_B_R antagonist), 0.43 to 7.87 μM CGP13501 (GABA_B_R-positive allosteric modulator), or the solvent control 0.4% dimethyl sulfoxide 60 min before behavior assessment at 5 days post fertilization. Co-exposures to positive allosteric modulators and PFOS were performed. Acute exposure to PFOS caused transient dark-phase hyperactivity. Concentration-dependent dark-phase hypoactivity was observed following acute propofol or CGP13501 exposure, in contrast to the concentration-dependent hyperactivity caused by acute picrotoxin exposure. Saclofen exposure provoked a modest reduction in dark-phase motor activity at the highest concentration tested. PFOS-induced hyperactivity was rescued to baseline activity by co-exposure to propofol or CGP13501. To assess relevance across species, electrophysiological measurements were performed in cultured mouse cortical neurons and BrainSpheres derived from human-induced pluripotent stem cells. PFOS exposure reduced GABA_A_R-mediated currents in mouse neurons. GABA_A_R- and GABA_B_R-dependent units in BrainSphere-derived neural networks exhibited increased spiking activity following PFOS exposure. This study demonstrates that PFOS antagonizes GABARs in zebrafish, mouse, and human experimental systems. Taken together, this study supports the concept that early life-stage zebrafish can be used to rapidly identify causative mechanisms, conserved across taxa, by which xenobiotic agents alter neuroactivity.

Per- and polyfluoroalkyl substances (PFAS) are a structurally diverse class of synthetic chemicals globally used in the manufacturing of industrial and consumer products (Wang et al. 2017). Nearly 15,000 PFAS have been identified by the US Environmental Protection Agency (US EPA) through systematic characterization of common carbon–fluorine substructures in combination with a percent fluorination threshold (US EPA PFASSTRUCTv5, last accessed December 2024; Gaines et al. 2023). Due to their surfactant-like, water-resistant, and flame-retardant properties (Kissa 2001; Krafft and Riess 2009), PFAS are widely distributed in water-repellent textiles and cosmetics, household chemicals, food packaging, non-stick cookware coatings, and firefighting foams (Herzke et al. 2012; Kotthoff et al. 2015; Whitehead et al. 2021). The prevalence of PFAS in conjunction with their innate chemical stability (Banks et al. 1994) and inadequate waste disposal methods (Stoiber et al. 2020) leads to persistent environmental contamination (Dreyer et al. 2009; Rankin et al. 2016), ultimately resulting in continuous exposure to humans (Sunderland et al. 2019; DeLuca et al. 2022) and wildlife (De Silva et al. 2021; Witt et al. 2024).

Perfluorooctanesulfonic acid (PFOS) is one long-chain, bioaccumulative PFAS that remains ubiquitous in drinking water (Xiao et al. 2015; Andrews and Naidenko 2020), air (Harrad et al. 2019), soil (Bräunig et al. 2019; Dauchy et al. 2019), humans (Pérez et al. 2013), and wildlife (Rupp et al. 2023). PFOS exposure is associated with a range of toxicity outcomes in both epidemiological studies and laboratory models, including developmental toxicity, immunotoxicity, neurotoxicity, and reproductive toxicity (Zeng et al. 2019; Fenton et al. 2021). To restrict the production and use of PFOS on a global scale, the 2009 Stockholm Convention on Persistent Organic Pollutants added PFOS to Annex B, which only allows limited use under specific exemptions (Stockholm Convention, Decision SC-4/17 2009). The National Primary Drinking Water Regulation passed by the US EPA in 2024 established legally enforceable limits on the levels of PFOS in drinking water (40 CFR 141 and 40 CFR 142, US EPA 2024). Despite a reduction in human serum PFOS levels since 1999 in the United States, the National Health and Nutrition Examination Survey (NHANES) reported that, in 2017 to 2018, PFOS was detected in 98% of serum samples with a mean concentration of 4.25 μg/l (CDC 2023). PFOS transfers through the placenta (Mamsen et al. 2019) and breast milk (Kärrman et al. 2007) and accumulates in the brain by penetrating the blood–brain barrier (Wang et al. 2018; Cao and Ng 2021). Additionally, prenatal and early life exposure is associated with behavioral deficits in children (Hoffman et al. 2010; Gump et al. 2011; Vuong et al. 2016; Kim et al. 2023; Zhang et al. 2024) and rodents (Butenhoff et al. 2009; Reardon et al. 2019; Mshaty et al. 2020), demonstrating the potential of PFOS to cause developmental neurotoxicity (DNT). There is, however, conflicting data on the DNT potential of PFAS, with some studies reporting inconsistent findings or no DNT effects in humans (Liew et al. 2018; Vuong et al. 2019; Forns et al. 2020; Skogheim et al. 2020).

Early life-stage zebrafish (*Danio rerio*) are a valuable vertebrate model for examining DNT. Along with rapid nervous system development and high genetic similarity to humans (∼70% orthologous genes) (Howe et al. 2013), behavior can be assessed as a functional readout of neurodevelopment directly following aqueous chemical exposure. Previous work in early life-stage zebrafish has illustrated that developmental or acute PFOS exposures cause behavioral alterations in the light–dark transition test (Gaballah et al. 2020; Menger et al. 2020; Rericha et al. 2021; Wu et al. 2022; Gutsfeld et al. 2024). In particular, PFOS-induced dark-phase hyperactivity at 5 days post fertilization (dpf) was shown to dissipate by 8 dpf, indicating a transient interaction between PFOS and a receptor rather than a perturbation in the neuronal circuit controlling dark-phase swimming behavior (Gutsfeld et al. 2024). There is limited evidence suggesting PFOS may interact with the gamma-aminobutyric acid receptor (GABAR), a primary inhibitory receptor of the central nervous system, to trigger hyperexcitation of a neuronal circuit. PFOS exposure inhibited GABA_A_R ion currents in *Xenopus* oocytes expressing a human GABA_A_R and evoked excitation of a rat cortical network (Tukker et al. 2020). Furthermore, zebrafish larvae without functioning GABA_A_R α_3_ and α_4/5_ subunits displayed hyperactive behavior at 48 hours post fertilization (hpf) (Barnaby et al. 2022). Based on these studies, we hypothesized that PFOS antagonizes GABARs to cause transient dark-phase hyperactivity.

To examine the molecular mechanism underlying the acute PFOS-induced phenotype in larval zebrafish, we first employed exposures to known pharmacological modulators of the ionotropic and metabotropic GABARs, GABA_A_R and GABA_B_R, respectively, in order to establish the behavioral profiles of chemicals with GABAergic action. We then performed co-exposures of PFOS and the pharmacological agents to determine whether GABA_A_R or GABA_B_R modulators can blunt or rescue dark-phase hyperactivity triggered by acute PFOS exposure. To demonstrate human relevance and verify GABAR interaction at the molecular level, we used electrophysiology approaches and pharmacological interventions targeting the GABARs across ex vivo and in vitro models. In cultured mouse cortical neurons, we conducted postsynaptic voltage-clamp recordings of GABA_A_R-mediated currents following PFOS exposure. Additionally, we exposed 3D BrainSphere neural networks derived from human-induced pluripotent stem cell (hiPSC)-based neural progenitor cells to PFOS and measured GABAR-mediated spontaneous electrical activity on microelectrode arrays (MEAs). Finally, to assess the possibility of PFOS binding the human α_1_β_2_γ_1_ GABA_A_R pore, we simulated docking of PFOS using the HADDOCK web server (Honorato et al. 2024). Building on the PFAS-induced behavioral phenotypes in larval zebrafish as described by Gaballah et al. (2020) and Gutsfeld et al. (2024), this study aimed to uncover the mechanism underlying PFOS-mediated behavioral hyperactivity, linking sulfonic acid PFAS exposure with a molecular initiating event conserved across taxa.

## Materials and methods 

### Zebrafish husbandry

All procedures involving zebrafish (*Danio rerio*) conducted at the Helmholtz Centre for Environmental Research (UFZ) were approved by Landesdirektion Sachsen (Geschäftszeichen 24-5131/252/7) and remained in line with German and European animal protection guidelines. Adult zebrafish of the strain TL were housed in 27 l glass tanks at a density of approximately 5 fish/l. The recirculating aquaculture system exchanged water at a rate of approximately 5×/h/tank with a 10% exchange from system to fresh water/day. Water is filtered and UV sterilized before circulating back into the tanks. The 14:10 light:dark cycle consisted of 8 h of direct overhead light and 6 h of ambient light to simulate fluctuations in the daily light levels. Water quality was assessed regularly based on the following parameters: water temperature: 26 to 29 °C; pH: 6 to 8; water hardness: 4 to 16°dH; nitrite: 0 to 1 mg/l; nitrate: 0 to 50 mg/l; ammonium: 0 to 0.35 mg/l. Adult zebrafish were fed twice daily, Monday to Friday, once with home-grown, shell-free artemia (Sanders) and once with Zebrafeed dry food (Sparos). On Saturday and Sunday, adult zebrafish were fed shell-free artemia (Sanders) once daily. For embryo collection, zebrafish were bred weekly by transferring 80 adults to 12 l sloping breeding tanks with a simulated shore area. The following morning, the off-rack adults were moved into new tanks, and embryos were collected 2 h later. Fertilized and morphologically normal embryos were selected using a dissection microscope (Olympus Szx7-ILLT).

### Chemical preparation

Heptadecafluorooctanesulfonic acid potassium salt (PFOS; Chemical Abstracts Service Registry No. (CASRN): 2795-39-3, Catalog No. 77282) and CGP13501 (CASRN: 56189-68-5, Catalog No. C0987) were purchased from Sigma-Aldrich. Picrotoxin (CASRN: 124-87-8, Catalog No. 1128) was purchased from Tocris Bioscience. Saclofen (CASRN: 125464-42-8, Catalog No. HY-100813) was purchased from MedChemExpress. 2,6-Diisopropylphenol (Propofol; CASRN: 2078-54-8, Catalog No. 683182) was purchased from HPC Standards.

Stock solutions (20, 40, or 60 mM) were prepared by dissolving the neat chemical into anhydrous dimethyl sulfoxide (DMSO; Sigma-Aldrich), and aliquots were stored at −80 °C. For single chemical exposures, one-use stock solution aliquots were thawed for each experiment and used to create 250× working solutions in line with previously published work (Gaballah et al. 2020; Gutsfeld et al. 2024). The 250× stock plate contained quarter-log serial dilutions of the chemical in DMSO in a 96-well polycarbonate microtiter plate, which was sealed and stored at room temperature (RT) in the dark before use. For co-exposure experiments, 500× working solutions were prepared.

### Study design and chemical exposure

In line with our previous work (Gaballah et al. 2020; Gutsfeld et al. 2024), embryos were bleached at 0 dpf using a 0.05% NaOCl solution. Bleached embryos were stored in glass crystallization dishes at a density of 1 embryo per 2 ml 10% Hanks’ Balanced Salt Solution (HBSS) at 28 °C until plating. At 1 dpf, single embryos with 400 µl of 10% HBSS were transferred to individual wells of a 96-square well clear polystyrene plate (Whatman Microplate Devices, Uniplate). To reduce evaporation and cross-contamination between wells, Microseal A film (Biorad MSA5001) was applied to the plate, which was then wrapped with parafilm. Plates were kept at 28 °C on a 14:10 h light:dark cycle until day 5. Unless otherwise indicated, for single chemical exposures, larvae underwent chemical exposure in the dark 60 min before behavior testing. For single chemical exposures, 1.6 µl of the 250× working solution was transferred to each well for a final concentration of 0.4% DMSO for all control and exposure groups. Following automated behavioral assessment, larvae were visually examined for survival, swim bladder inflation, and general malformations such as edemas, body axis defects, and structural abnormalities. If larvae were dead, malformed, or possessed an uninflated swim bladder, they were excluded from behavioral analysis.

For co-exposure experiments, a 500× working solution of both PFOS and the GABAR modulator was created. 75 min before behavior testing, 0.8 µl of the working solution for the GABAR modulator was transferred to each well, resulting in a concentration of 0.2% DMSO. 15 min later, 0.8 µl of the working solution for PFOS was applied, leading to a final concentration of 0.4% DMSO. Behavior was assessed 60 min following PFOS exposure.

#### Acute PFOS exposure

To reproduce PFOS-induced acute neurotoxicity as previously outlined (Gutsfeld et al. 2024), zebrafish larvae were exposed to 7.87 to 120 µM PFOS 60 min before behavior testing at 5 dpf in the light–dark transition test. Replicate numbers ranged from 21 to 45. From these concentration-response experiments, 120 µM PFOS was selected for future exposures, as it produced significant dark-phase hyperactivity. The time-of-peak-effect for PFOS was determined by exposing larvae to 120 µM PFOS at 30, 60, 120, or 240 min before measuring behavior. Replicate numbers ranged from 45 to 48.

#### Acute GABAR modulator exposure

To determine whether the acute PFOS dark-phase hyperactivity phenotype could be phenocopied, larvae were exposed to 0.68 to 12.4 µM of the GABA_A_R antagonist picrotoxin or 4.4 to 80 µM of the GABA_B_R antagonist saclofen. To select GABAR modulators for rescue experiments, 0.77 to 14.05 µM of the GABA_A_R-positive allosteric modulator (PAM) propofol or 0.43 to 7.87 µM of the GABA_B_R PAM CGP13501 were applied. All exposures were performed 60 min before behavior assessment. Replicate numbers ranged from 13 to 48.

#### Co-exposures

To examine whether the PFOS hyperactivity phenotype could be rescued by a GABAR PAM, demonstrating that PFOS functions as a GABAR antagonist in zebrafish, the GABA_A_R and GABA_B_R PAMs, propofol, and CGP13501 were selected. Larvae were exposed to 0.43 µM propofol or 1.38 µM CGP13501, both concentrations that caused an intermediate hypoactivity phenotype in the acute GABAR modulator exposures. 15 min later, 120 µM PFOS was applied, and behavior assessment was initiated 60 min later. Replicate numbers ranged from 65 to 72.

### Automated behavior assay

On the morning of behavior assessment, 96-well plates containing larvae were placed in boxes in the incubator to prevent any light exposure on the day of testing. Plates were exposed to chemical solution and transferred to the behavior apparatus (Zebrabox, Viewpoint) in the dark under red light. The light–dark transition test protocol was comprised of a 20 min dark acclimation at 0 lux, 20 min light at 13,238 lux (light phases L1 and L2), and 22 min dark at 0 lux (dark phases D1 and D2) phase. Zebrafish locomotion was captured on video using an infrared camera at 25 frames/s. Locomotor activity was quantified by Zebralab Software (Viewpoint) in the tracking algorithm, in which data were obtained at 1s intervals.

#### Statistics and data visualization

The entire pipeline for data analysis and visualization is available as a user-friendly set of functions (v0.1, https://doi.org/10.5281/zenodo.11396730).

As described in Gutsfeld et al. (2024), for phase data analysis, the individual distance moved per larvae was summarized in 2min periods. For each assay phase (L1, L2, D1, D2), five 2min-sums per lava were calculated. A Generalized Additive Mixed Effects Model (GAMM [Wood 2017]) was used to fit the data, and all variables are described here. Distance moved was fitted using a beta distribution calculated as 1.001 times the maximum distance moved per larva within an experiment. A beta distribution was chosen because distances are bound by zero and a maximum. The nonlinear effect of time was modeled using smoothing splines. Concentration and phase were modeled as categorical variables, and their second-order interactions were included. Variability between individual larvae was modeled as random effects because all individuals were repeatedly measured. The GAMM was applied using the R package mgcv.

The model formula was as follows:


Logit(distance moved)∼ s(time)+concentration+phase+concentration:phase+(1|animal)+ϵ

where:

-
logit(distance moved) : distance moved scaled from 0 to 1, and logit transformed
-
s(time) : smoothing spline for the trend through time, corrected for autocorrelation
-
concentration : categorical variable, the different concentrations tested
-
phase : categorial variable, the different assay phases
-
concentration:phase : second-order interaction of concentration and assay phase
-
(1|animal) : random effect, to control for variability between larvae and repeated measures design
-
ϵ : error term following a beta distribution

To assess the model quality, residuals and fitted smooths were visually inspected. To obtain *P*-values, estimated marginal means (EMMs) were calculated based on the fitted model as post hoc tests using the R package emmeans. A Tukey-adjust was performed on the obtained *P*-values to account for multiple comparisons.

For visual startle response (VSR) data analysis, the VSR was first calculated as the summed distance moved in the 3 s following dark–light (VSR1) or light–dark (VSR2) transitions. The VSR was modeled using a linear mixed effects model with the R package lme4.

The model formula was as follows:


startle response ∼ concentration+startle phase+(1|animal)+ϵ

where:

-
startle response: startle response measured
-
concentration: categorial variable, the different concentrations tested
-
startle phase: categorial variable, either VSR1 or VSR2
-
(1|animal): random effect, to control for variability between larvae and repeated measures design
-
ϵ: error term following a Gaussian distribution

To obtain *P*-values, EMMs were calculated based on the fitted model as post hoc tests using the R package emmeans. A Tukey-adjust was performed on the obtained *P*-values to account for multiple comparisons.

All statistical analyses and visualizations were done using custom-built scripts in R (version 4.3.1; R Development Core Team) and the following packages:

- reshape2 (v.1.4.4 [Wickham 2010])
- ggplot2 (v.3.3.4 [Wickham et al. 2007])
- car (v.3.1-3 [Fox et al. 2001])
- dplyr (v.1.1.4 [Wickham et al. 2014])
- data.table (v.1.14.8 [Barrett et al. 2006])
- openxlsx (v.4.2.5.2 [Schauberger and Walker 2014])
- multcompView (v.0.1-9 [Graves et al. 2006])
- lme4 (v.1.1-35.5 [Bates et al. 2003])
- emmeans (v.1.10.5 [Lenth 2017])
- tidyverse (v.2.0.0 [Wickham 2016])
- mgcv (v.1.8-42 [Wood 2000])

### GABA quantification in zebrafish

#### Sample preparation

Zebrafish were bred, and embryos were collected and bleached as described above. Bleached embryos were stored in glass crystallization dishes at a density of 1 embryo per 2 ml 10% HBSS at 28 °C. At 4 dpf, dishes were examined, and coagulated or malformed embryos were removed. For sample collection at 5 dpf, larvae were anesthetized by placing the dishes in ice-cold 10% HBSS for ≥20 min, then groups of 40 larvae were placed into 2 ml tubes (Eppendorf Tubes) filled with metal beads (1 mm) for homogenization. Excess media were removed, and larvae were flash-frozen in liquid nitrogen and stored at −80°C until use.

#### Metabolomics

Each sample was mixed with 100 µl of the extraction solvent acetonitrile (ACN):H_2_O (1:1, v/v) and homogenized using a TissueLyser II (30 Hz, 10 min; Retsch Qiagen). After centrifugation (2 min, 14,000 rpm), 10 µl were evaporated to dryness (SpeedVac, Eppendorf). For derivatization of amino acids and biogenic amines, the samples were resuspended in 50 µl 5% phenyl isothiocyanate (PITC) in ethanol:H2O:pyridine (1:1:1, v/v/v) and incubated for 25 min at RT. Subsequently, samples were dried to remove excess PITC and resuspended in 10 µl 5 mM ammonium acetate in methanol. After incubation (10 min, 14,000 rpm), 90 µl H_2_O:ACN + 0.2% formic acid were added. Prior to measurement, 10 µl of each derivative was injected onto a Waters Acquity UPLC system coupled on-line with a QTRAP 5000 mass spectrometer (Sciex, Framingham, USA). Chromatographic separation was achieved with an Agilent Zorbax Eclipse XDB-C18 column (3.5 µm, 3.0 × 100 mm) using a constant flowrate of 0.5 ml/min and water + 0.2% formic acid and ACN + 0.2% formic acid as mobile phases A and B, respectively. The linear LC gradient was as follows: 0 to 0.5 min at 0% B, 0.5 to 4 min 0% to 70% B, 4 to 5.3 min 70% B, 5.3 to 5.4 min 70% to 0% B, 5.4 to 7.3 min 0% B, and the QTRAP was set up to positive ionization mode. For identification and quantitation, a scheduled multiple reaction monitoring (MRM) method was used, with specific transitions for every metabolite. External calibration curves for each metabolite were measured for linear regression. Peak areas of all samples and standards for linear regression were determined in SciexOS Software (v. 3.0.0., Sciex).

### Mouse cortical network assay

#### Mouse cortical cultures

Neocortical neuronal cultures from P0 to P1 mice were prepared as described previously (Moutin et al. 2020). Briefly, mice were decapitated, and cerebral cortices were removed, dissected, and enzymatically digested with papain (Sigma, CASRN: 9001-73-4) or trypsin (Sigma, CASRN: 9007-07-7) in the presence of DNAse (Sigma, CASRN: 9003-98-9), followed by mechanical dissociation and centrifugation through a cushion of 4% bovine serum albumin (Sigma, CASRN: 9048-46-8). These steps were completed using Hibernate medium (ThermoFisher). Cells were then plated onto Poly-L-Lysine (Sigma, CASRN: 9001-73-4)-coated coverslips in 24-well plates. For each coverslip, 25 to 30 k cells were allowed to settle in a 40 μl drop for approximately 30 min, and then each well was filled with 500 μl of NeurobasalA/B27 growth medium (Invitrogen) supplemented with GlutaMax (0.25%, Invitrogen), glutamine (0.25 to 0.5 mM, Sigma), penicillin/streptomycin (1:100, ThermoFisher), and heat-inactivated fetal calf serum (10%, Sigma, CASRN: 1943609-65-1). Media was partially exchanged on day 3 (800 μl) and day 7 (500 μl) with fresh maintenance medium comprised of BrainPhys (StemCell), B27 (2%, Invitrogen), GlutaMax (0.25%, Invitrogen), and penicillin/streptomycin (1%, ThermoFisher). Cultures were maintained for up to 2 to 3 weeks at 37 °C and 5% CO_2_ until use.

#### Electrophysiology

Postsynaptic voltage-clamp recordings were performed at mouse cortical neurons (days in vitro 13 to 14] using a HEKA EPC10 amplifier (HEKA Elektronik, Lambrecht/Pfalz, Germany). Pipette solution for voltage clamp recordings contained (in mM): 120 CsCl, 20 TEA-Cl, 10 HEPES, 5 EGTA, 3 Mg-ATP, 0.3 Na-GTP, 5 Na-Phosphocreatin, 3 QX314-Cl, pH adjusted with CsOH to 7.3, and osmolarity adjusted with sucrose to 292 mOsm. Series resistance (Rs) was on average at 8.8 ± 2.7 MΩ. Pipettes were pulled from borosilicate glass (Science Products, Hofheim, Germany) with a DMZ Universal Electrode (Zeitz Instruments, Martinsried, Germany) with a resistance of 3 to 4 MΩ. Recordings of the holding current were performed at a holding potential of −70 mV in extracellular Tyrode’s solution containing (in mM): 145 NaCl, 2.5 KCl, 1.2 MgCl_2_, 2 CaCl_2_, 10 HEPES, 10 glucose, pH adjusted by NaOH to 7.4. To isolate GABA_A_R-mediated currents, the extracellular solution was supplemented by 10 µM NBQX (Biotechne, CASRN: 479347-86-9), 50 µM APV (Tocris, CASRN: 79055-68-8), and 3 µM CGP55845 (Biotrend, CASRN: 49184-22-5) to block AMPAR, NMDAR, and GABA_B_R, respectively. A junction potential of 2 mV was not corrected. Recordings were performed at RT.

After establishing a baseline recording period (before condition ∼3 min), 50 µM GABA was washed in through the perfusion system, and the holding current was monitored for 4 min, representing a GABA_A_R-mediated current (IGABA) in recording solution (control) and recording solution supplemented by 120 µM PFOS (+PFOS). PFOS was washed in through the perfusion system 5 to 10 min prior to GABA application.

#### Statistics and data visualization

Postsynaptic currents were analyzed with the NeuroMatic plug-in44 (Version 3) for Igor Pro (WaveMetrics, Lake Oswego, OR, USA; Version 9). The obtained parameters were presented with Sigma Plot 11 (Systat Software). To evaluate the effect of PFOS on GABA_A_R-mediated currents, the maximum changes of the holding current in cells measured under control conditions, and cells under PFOS perfusion were statistically compared with a Mann–Whitney *U*-test with jamovi (https://www.jamovi.org).

### Human multi-neurotransmitter receptor assay

#### Human cell cultures

The human multi-neurotransmitter receptor (hMNR) assay uses 3D BrainSpheres derived from hiPSC-based neural progenitor cells (hiNPCs). HiPSCs (line IMR90, clone 4, WiCell, Madison, WI, USA) were cultured and neurally induced following the 2D-NIM protocol, described by Hartmann et al. (2023). Briefly, hiPSC colonies were singularized and cultured in a defined medium, including the dual SMAD inhibitors SB-431542 and LDN-193189 on pre-coated (Polyethyleneimine, Laminin LN521) six-well plates for 21 days in 2D. After 21 days, hiNPCs were singularized and cryopreserved as a single-cell suspension. For sphere formation, hiNPCs were thawed, transferred to six-well plates, and cultivated in a gyrical shaking incubator (140 rpm, 12.5 mm diameter) in neural progenitor medium for 7 days, followed by transfer to proliferation medium (Hartmann et al. 2023). HiNPC spheres (0.3 mm in diameter) were differentiated in 3D in CINDA+ differentiation medium (Nimtz et al. 2020; Hartmann et al. 2023) for 3 weeks with half-medium exchanges three times per week. After 3D differentiation, BrainSpheres were plated on pre-coated (Poly-L-Ornithine, Laminin LN521) 96-well MEAs (#M768-tMEA-96B, Axion Biosystems) in CINDA+ differentiation medium (1 sphere per well) and cultured for 4 weeks with half-medium exchanges 3 times per week.

#### MEA recordings

After 4 weeks of differentiation on the MEAs, spontaneous electrical baseline activity was recorded. All extracellular recordings of electrical activity were performed for 15 min at 37 °C and 5% CO_2_ in the Axion Maestro Pro System (Axion Biosystems) according to Hartmann et al. (2023). For initial GABAergic unit identification, BrainSpheres were acutely exposed to the neurotransmitter γ-aminobutyric acid (GABA; 10 µM). After electrical activity was measured, neurotransmitter receptor antagonists were added to each well (10 µM bicuculline for GABA_A_Rs, 5 µM saclofen for GABA_B_Rs), network activity was again recorded, and substances were removed with a complete exchange of the medium. After a 3 h washout performed by complete exchange of media, the second baseline was recorded, and PFOS was gradually added at sequentially increasing concentrations (7.78, 14.05, 25.09, 44.80, 80, 100, and 120 µM). For further analysis of detected spikes, recorded AxIS .spk files were concatenated in the order of measurement and converted into a single .nex file with a MATLAB (R2021b, R2022b, MathWorks, Natick, MA, USA) script. Spike sorting of the .nex file was performed using the Offline Sorter (OFS, version 4.4, Plexon, Dallas, TX, USA) software, applying the automatic clustering T-Distribution EM method (10 degrees of freedom (D.O.F), 20 initial units).

#### Statistics and data visualization

Data analysis was performed using a custom R-script (version 4.3.1; R Development Core Team) with the PMCMRplus (v 1.9.12 [Pohlert 2018], statistical analysis) and ggplot2 (v.3.3.4 [Wickham et al. 2007], data visualization) packages. For each baseline recording, data were filtered to exclude non-firing or inactive units. Specifically, only entries with spike counts >0 in both the initial baseline (recording 1) and the second baseline (recording 4) were retained. The 2.5th and 97.5th percentiles were then calculated to provide a non-parametric estimate of the 95% confidence interval bounds. Based on this, recordings with spike counts between ≥3 and ≤1601 in the initial baseline (recording 1) and between ≥2 and ≤2327 in the second baseline (recording 4) were included in the analysis (Fig. S9).

To determine neuronal subtype-specific responses, units were classified as either GABA_A_R- or GABA_B_R-dependent based on their spiking activity in response to neurotransmitter and receptor antagonist treatments. A unit was classified as GABA_A_ if spiking decreased in response to GABA (GABAR agonist) treatment (recording 2) relative to initial baseline (recording 1), and subsequently increased in response to bicuculline (GABA_A_R antagonist) treatment (recording 3) relative to GABA treatment. GABA_B_ units were similarly identified but with the application of saclofen (GABA_B_R antagonist) in recording 3. For both neuronal subtypes, PFOS exposure conditions were mapped to recording segments 5 to 11, corresponding to PFOS concentrations ranging from 7.78 to 120 µM. To account for baseline variability in spiking activity across units, spike counts were normalized to the total spike count per unit across all analyzed recording segments, ensuring comparability across conditions.

A Friedman test for paired data was conducted to compare normalized spike counts at each PFOS concentration against baseline (0 µM) within the same unit. Post hoc analysis was performed using the Conover test. To control for false discovery rate, *P*-values from comparisons against the control condition (0 µM) were adjusted using the Benjamini–Hochberg method.

### Ligand docking with the HADDOCK 2.4 web server

Docking calculations were performed using the HADDOCK (High Ambiguity Driven Docking) 2.4 web server (Honorato et al. 2021, 2024). Default settings for ligand–protein binding were applied, with the RMSD cutoff for clustering set to 2.0 Å. The human α_1_β_2_γ_1_ GABA_A_ receptor structure used for docking is derived from PDB ID: 8SG0 (Legesse et al. 2023). Using the PDB-tools software package, all pre-bound ligands contained in the structures were removed, and the residue numbering was changed to meet HADDOCK input requirements (Rodrigues et al. 2018). The structure of PFOS was isolated from PDB ID: 4E99 by applying PDB-tools to retain only the ligand coordinates (Luo et al. 2012). Using a hypothesis-driven approach, pore-lining amino acids were selected to guide the docking of PFOS into the GABA_A_R pore: α_1_ (P253, V257, T261, L264), β_2_ (A248, A252, T256, L259), and γ_2_ (P263, S267, I270, T271). For the initial rigid-body docking, the aforementioned amino acids, as well as PFOS, were defined as active participants to draw PFOS within the GABA_A_R pore. In the subsequent flexible refinement stages, the restraints were defined as passive and PFOS as active, allowing PFOS to explore the restrained channel pore.

## Results

### Acute PFOS exposure induces dark-phase hyperactivity in larval zebrafish

**Fig. 1.**
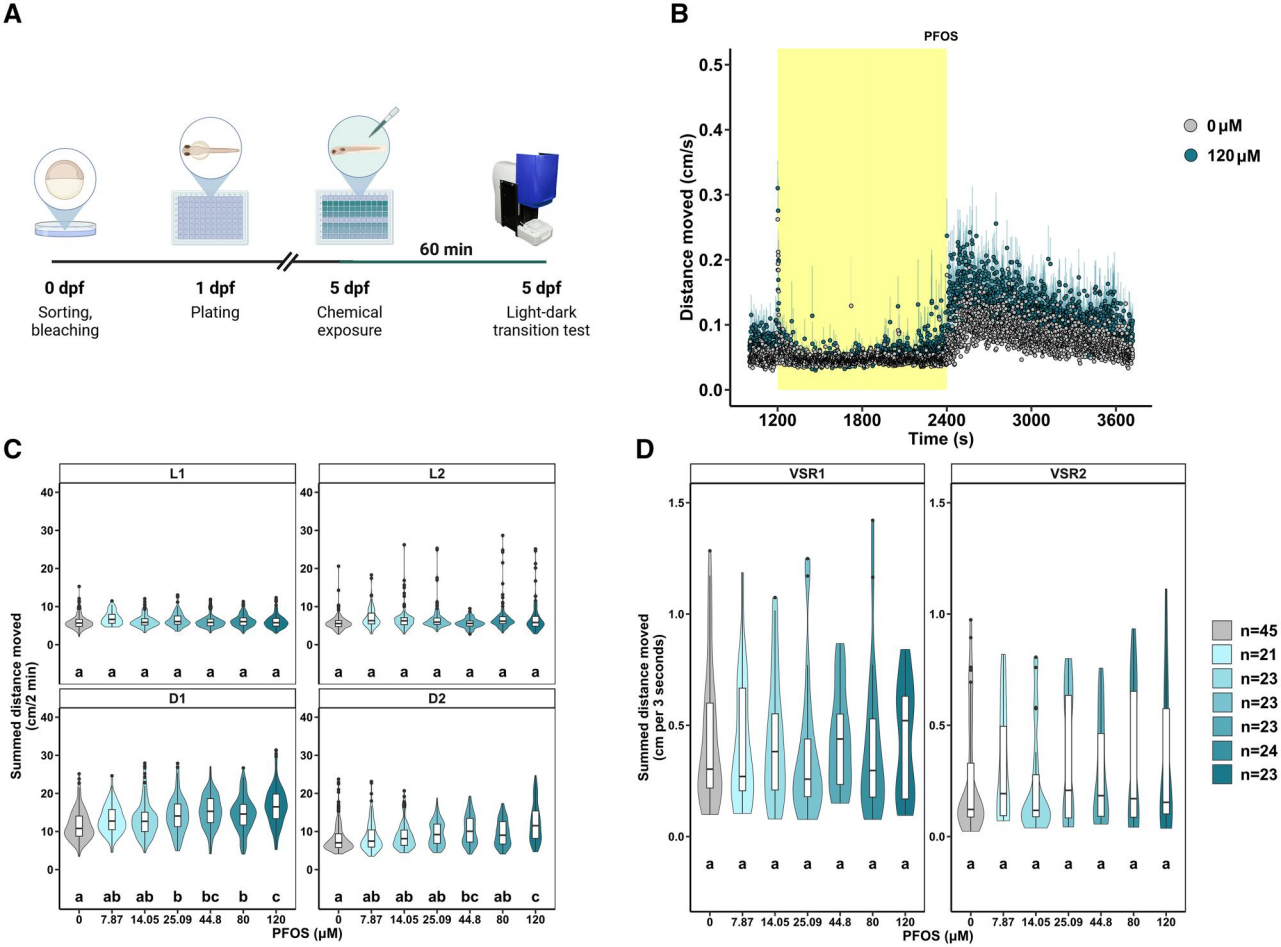
Dark-phase hyperactivity is observed in 5 dpf zebrafish larvae acutely exposed to PFOS. (A) Schematic representation of the exposure paradigm in which 5 dpf larvae were exposed to 7.87 to 120 µM PFOS or 0.4% DMSO as a vehicle control 60 min before behavioral assessment. (B) Locomotor activity over time following exposure to 120 µM PFOS (blue; *n* = 23) or 0.4% DMSO (grey; *n* = 45) in the light phase (yellow; 1200 to 2400 s) at 13,238 lux and dark-phase (white; 2400 to 3720 s) at 0 lux of the light–dark transition test. Data are mean distance moved per second (cm/s) ± standard error. (C) Box- and violin-plots of motor activity in each 10 min assay phase (L1, L2, D1, D2) following 7.87 to 120 µM PFOS (*n* = 21 to 24) exposure or 0.4% DMSO (*n* = 45). Data are summed distance moved in 2 min intervals (cm/2 min) for each larva. (D) Box- and violin plots of motor activity (cm) in the 3 s following the dark–light (VSR1) or light–dark (VSR2) transition following 7.87 to 120 µM PFOS (*n* = 21 to 24) exposure or 0.4% DMSO (*n* = 45). Boxes indicate the median and interquartile range (IQR), whiskers indicate the calculated minimum (25th percentile −1.5 × IQR) and the calculated maximum (75th percentile +1.5 × IQR), and dots indicate the outliers beyond the calculated minima and maxima. Violins describe the kernel probability density of the underlying data. Significance (*P *< 0.05) is displayed as different letters and was determined by Tukey-adjusted estimated marginal means following either a (C) generalized additive mixed effects model (Fig. S1) or a (D) linear mixed effects model. Summary data are located in Excel Tables S1 to S3 (Owen 2025a). dpf, days post fertilization; L, light; D, dark; DMSO, dimethyl sulfoxide; PFOS, perfluorooctanesulfonic acid; VSR, visual startle response; IQR, interquartile range.

To investigate how PFOS interacts with a receptor to elicit transient neurotoxicity, we acutely exposed zebrafish larvae at 5 dpf to 7.87 to 120 µM PFOS for 60 min and assessed locomotor activity in a light–dark transition test (Fig. 1A and B). Acute PFOS exposure caused significant hyperactivity in the D1 (25.09 to 120 µM) and D2 (44.8, 120 µM) phases (Fig. 1C), as defined by a statistically significant increase in motor activity following PFOS exposure compared with the DMSO control. Acute PFOS exposure did not affect startle response activity in the light–dark (VSR1) or dark–light (VSR2) transition (Fig. 1D). We selected 120 µM PFOS for future exposures as it caused pronounced hyperactivity in the dark phase (Fig. 1B and C).

**Fig. 2.**
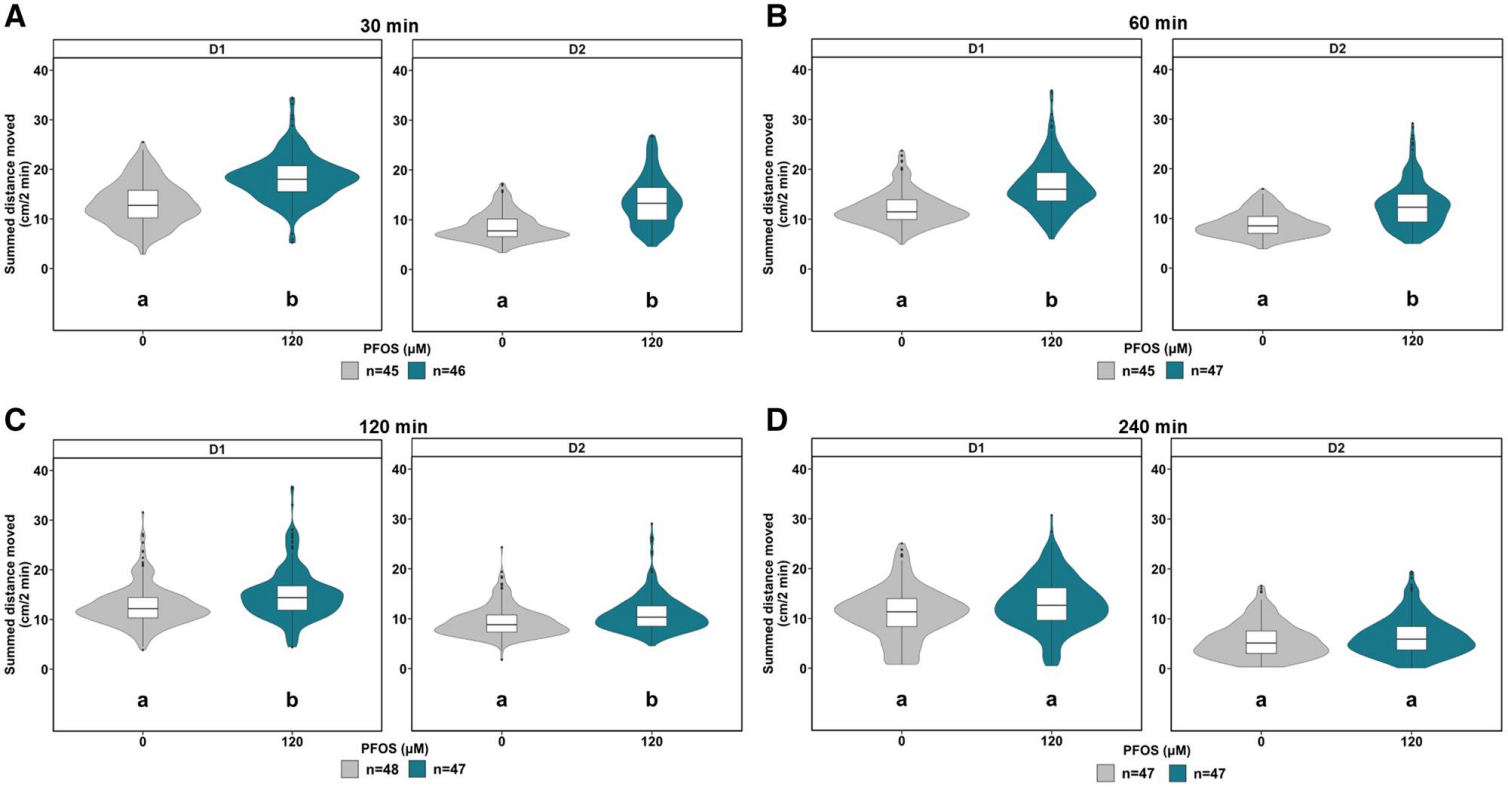
The optimal PFOS exposure window to trigger dark-phase hyperactivity in 5 dpf zebrafish is between 30 and 60 min. Box- and violin-plots signifying the distance moved (cm) in 2 min periods across the first 10 min in the dark (D1) and the next 10 min in the dark (D2) at 0 lux for each larva exposed to 120 µM PFOS (blue) or 0.4% DMSO (grey) for (A) 30 min, (B) 60 min, (C), 120 min, or (D) 240 min before locomotor assessment. Replicate numbers range from 45 to 47 larvae per test group. Light phase and visual startle response activity can be found in the supplement (Fig. S2). Boxes indicate the median and interquartile range (IQR), whiskers indicate the calculated minimum (25th percentile −1.5 × IQR) and the calculated maximum (75th percentile +1.5 × IQR), and dots indicate the outliers beyond the calculated minima and maxima. Violins describe the kernel probability density of the underlying data. Significance (*P *< 0.05) is displayed as different letters and was determined by Tukey-adjusted estimated marginal means following a generalized additive mixed effects model (Fig. S3). Summary data are located in Excel Tables S4 to S13. D, dark; DMSO, dimethyl sulfoxide; PFOS, perfluorooctanesulfonic acid; IQR, interquartile range.

To determine the optimal exposure window to observe PFOS-dependent dark-phase hyperactivity, larvae were exposed to 120 µM PFOS at 30, 60, 120, or 240 min before behavioral assessment (Fig. 2). D1 hyperactivity peaked between 30 and 60 min PFOS exposure (Fig. 2A and B), and locomotor activity returned to baseline by 240 min PFOS exposure (Fig. 2D), highlighting the transient nature of the phenotype. Based on the observed time-of-peak-effect, the window of PFOS exposure remained at 60 min for subsequent experiments.

### Pharmacological agents targeting GABARs modulate dark-phase swimming behavior

**Fig. 3.**
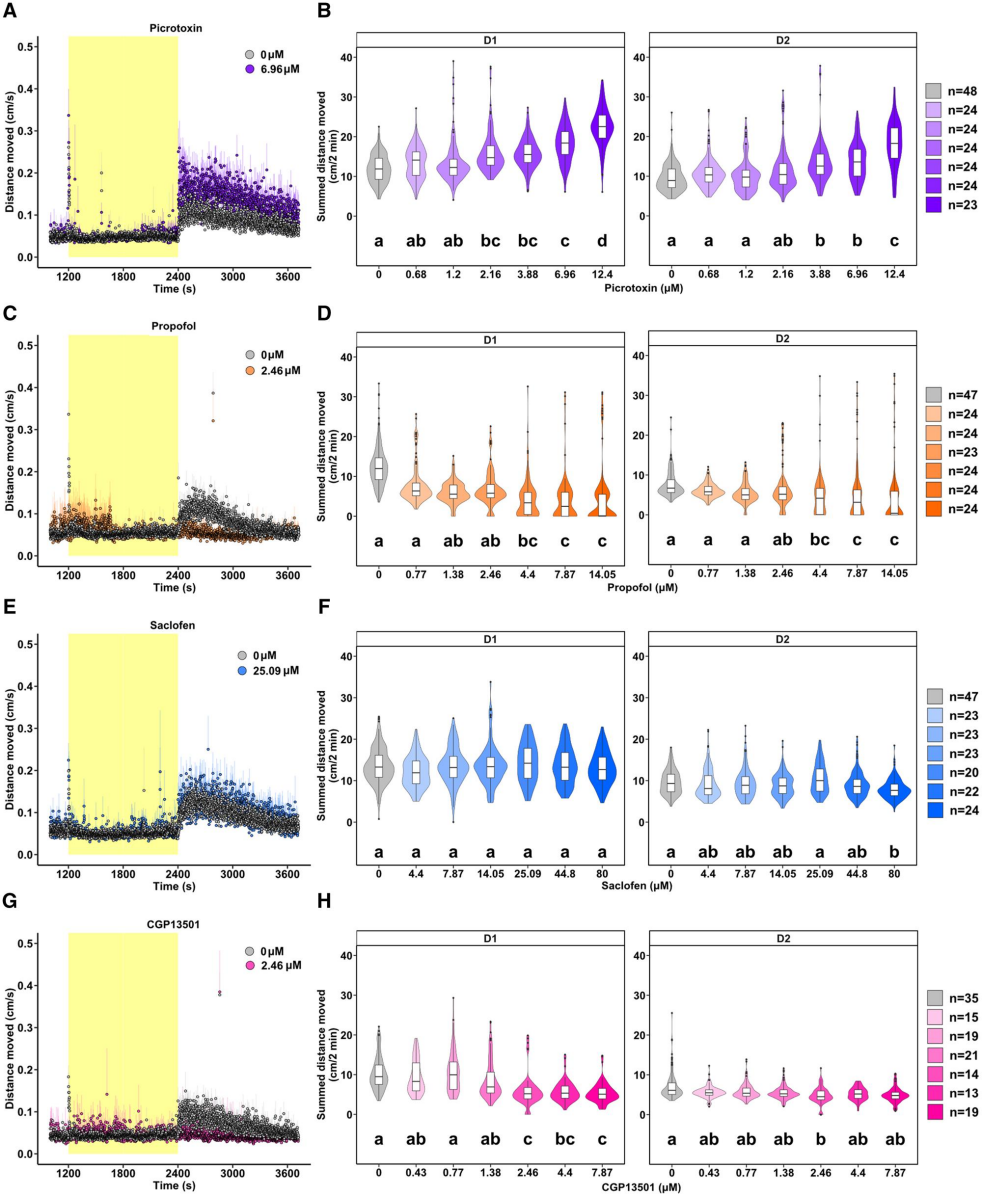
Exposure to GABAR modulators picrotoxin, propofol, and CGP13501, but not saclofen, causes changes in swimming behavior at 5 dpf. Representative time series of locomotor activity of larvae exposed to (A) 6.96 µM GABA_A_R antagonist picrotoxin (purple; *n* = 24), (C) 2.46 µM GABA_A_R PAM propofol (orange; *n* = 23), (E) 25.09 µM GABA_B_R antagonist saclofen (blue; *n* = 20), or (G) 2.46 µM GABA_B_R PAM CGP13501 (pink; *n* = 14) compared with 0.4% DMSO control (grey; *n* = 35-48). Data are mean distance moved per second (cm/s) ± standard error across the light phase (yellow; 1200 to 2400 s) at 13,238 lux and dark phase (white; 2400 to 3720 s) at 0 lux of the light–dark transition test. Box- and violin-plots of motor activity in the D1 and D2 phases following exposure to (B) 0.88 to 12.4 µM picrotoxin, (D) 0.77 to 14.05 µM propofol, (F) 4.4 to 80 µM saclofen, or (H) 0.43 to 7.87 µM CGP13501. Light-phase data can be found in the supplement (Fig. S5). Data are summed distance moved in 2 min intervals (cm/2 min) for each larva. Boxes indicate the median and interquartile range (IQR), whiskers indicate the calculated minimum (25th percentile −1.5 × IQR) and the calculated maximum (75th percentile +1.5 × IQR), and dots indicate the outliers beyond the calculated minima and maxima. Violins describe the kernel probability density of the underlying data. Significance (*P *< 0.05) is displayed as different letters and was determined by Tukey-adjusted estimated marginal means following a generalized additive mixed effects model (Fig. S6). Summary data are located in Excel Tables S14 to S18. GABAR, GABA receptor; dpf, days post fertilization; D, dark; DMSO, dimethyl sulfoxide; IQR, interquartile range.

We hypothesized that PFOS antagonizes GABARs, thereby blocking their inhibitory input to the neuronal circuitry controlling dark-phase swimming behavior, resulting in hyperactivity. To explore the role of the GABARs in larval zebrafish locomotion, we first quantified the levels of GABA to confirm that GABA is detectable in 5 dpf zebrafish (Fig. S4). We then characterized the neuroactivity and behavioral profiles of a suite of pharmacological agents that modulate GABA_A_R and GABA_B_R. The GABA_A_R antagonist picrotoxin caused concentration-dependent hyperactivity in the D1 (2.16 to 12.4 µM) and D2 (3.88 to 12.4 µM) endpoints (Fig. 3A and B), demonstrating that picrotoxin phenocopies the behavioral response induced by PFOS. Acute exposure to GABA_A_R or GABA_B_R PAMs, propofol and CGP13501, respectively, triggered behavioral hypoactivity across multiple concentrations. Propofol induced hypoactivity in the D1 (4.4 to 14.05 µM) and D2 (4.4 to 14.05 µM) phases (Fig. 3C and D). Larvae exposed to 2.46 to 7.87 µM CGP13501 displayed hypoactivity in the D1 phase, whereas only exposure to 2.46 µM caused hypoactivity in D2 (Fig. 3G and H). The GABA_B_R antagonist saclofen failed to evoke behavior changes in D1 at the concentrations tested (4.4 to 80 µM), although 80 µM exposure produced a modest yet significant reduction in activity in the D2 phase (Fig. 3E and F). The behavioral changes observed with the application of these pharmacological agents suggest that GABAR modulation functions as a switch for dark-phase motor activity.

### PAMs of GABARs rescue the PFOS-triggered phenotype

**Fig. 4.**
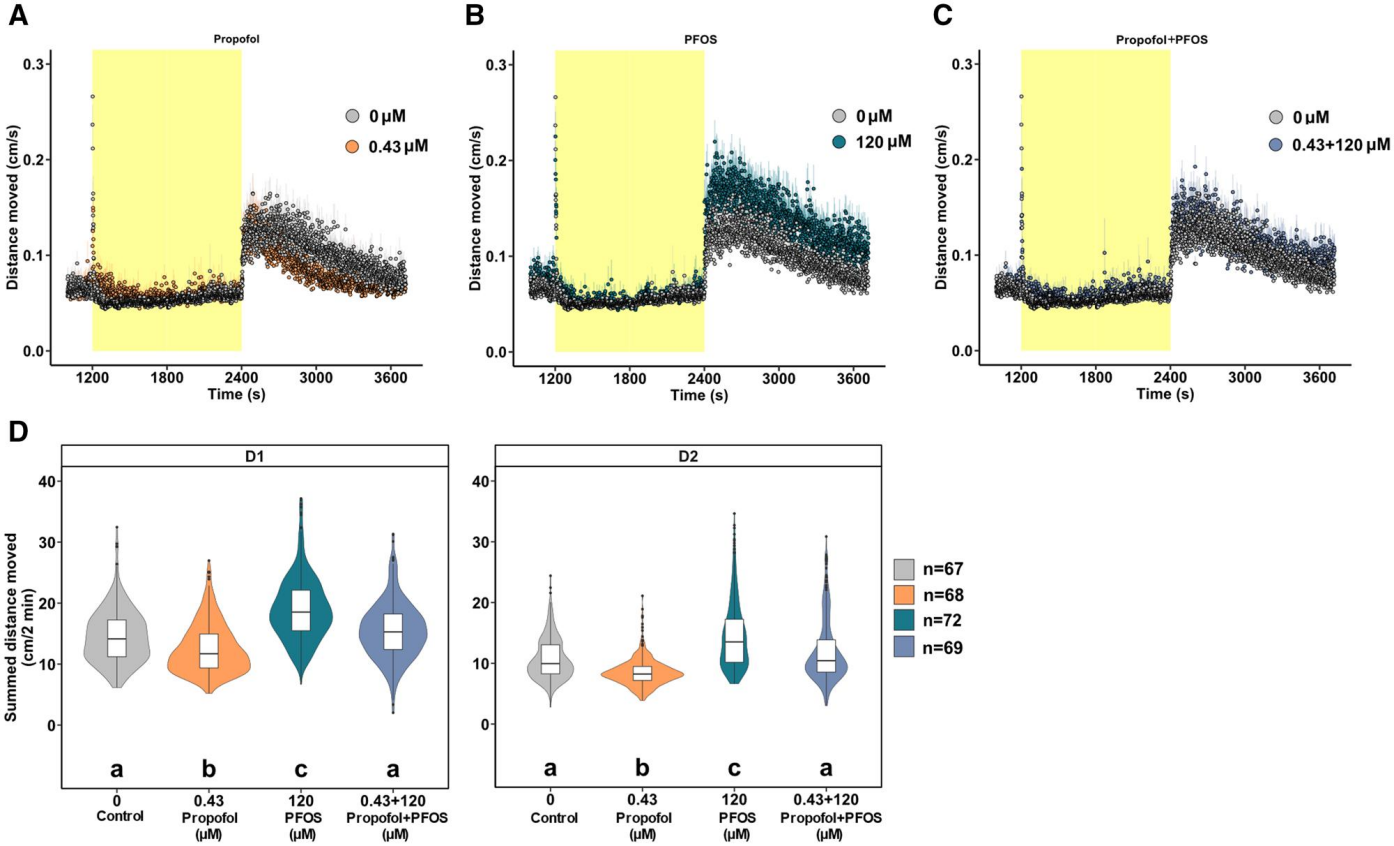
Co-exposure to the GABA_A_R PAM propofol and PFOS blunts dark-phase hyperactivity to control levels. Locomotor activity of 5 dpf larvae exposed to (A) 0.43 µM GABA_A_R PAM propofol (orange; *n* = 68), (B) 120 µM PFOS (blue; *n* = 72), or (C) propofol and PFOS (lavender-blue; *n* = 69) compared with 0.4% DMSO control (gray; *n* = 67). Data are mean distance moved per second (cm/s) ± standard error across the light phase (yellow; 1200 to 2400 s) at 13,238 lux and dark phase (white; 2400 to 3720 s) at 0 lux of the light–dark transition test. Box- and violin-plots of summed distance moved in 2 min intervals (cm/2 min) for each larva in the (D) D1 phase and D2 phase. Light-phase data can be found in the supplement (Fig. S7). Boxes indicate the median and interquartile range (IQR), whiskers indicate the calculated minimum (25th percentile −1.5 × IQR) and the calculated maximum (75th percentile +1.5 × IQR), and dots indicate the outliers beyond the calculated minima and maxima. Violins describe the kernel probability density of the underlying data. Significance (*P *< 0.05) is displayed as different letters and was determined by Tukey-adjusted estimated marginal means following a generalized additive mixed effects model (Fig. S8). Summary data are located in Excel Tables S19 and S20. GABA_A_R, GABA_A_ receptor; PFOS, perfluorooctanesulfonic acid; PAM, positive allosteric modulator; dpf, days post fertilization; D, dark; DMSO, dimethyl sulfoxide; IQR, interquartile range.

**Fig. 5.**
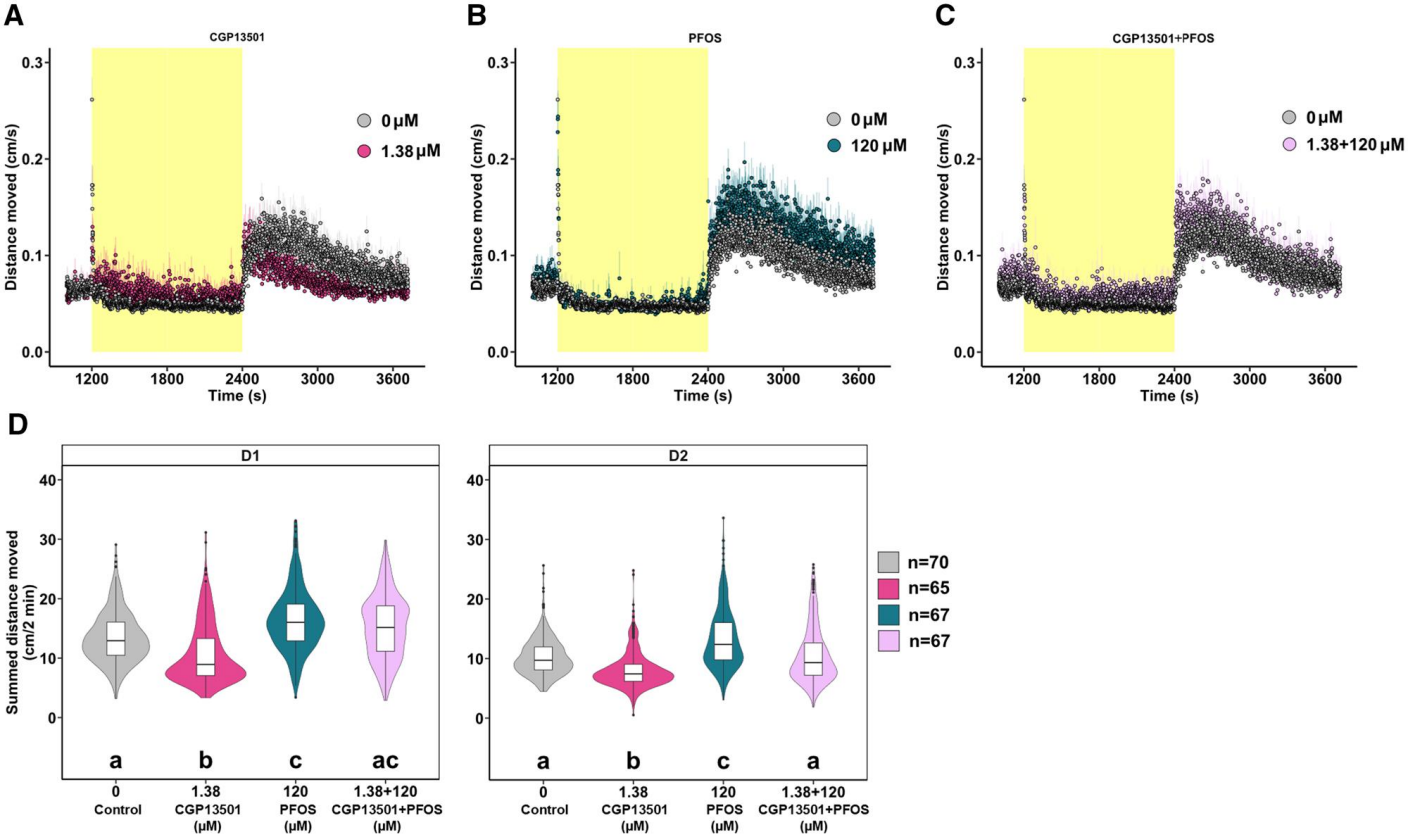
Dark-phase hyperactivity is rescued by GABA_B_R PAM CGP13501 and PFOS co-exposure. Locomotor activity of 5 dpf larvae exposed to (A) 1.38 µM GABA_B_R PAM CGP13501 (pink; *n* = 65), (B) 120 µM PFOS (blue; *n* = 67), or (C) the co-exposure of CGP13501 and PFOS (lilac; *n* = 67) compared with 0.4% DMSO control (grey; *n* = 70). Data are mean distance moved per second (cm/s) ± standard error across the light phase (yellow; 1200 to 2400 s) at 13,238 lux and dark-phase (white; 2400 to 3720 s) at 0 lux of the light–dark transition test. Box- and violin-plots of summed distance moved in 2 min intervals (cm/2 min) for each larva in the (D) D1 phase and D2 phase. Light-phase data can be found in the supplement (Fig. S7). Boxes indicate the median and interquartile range (IQR), whiskers indicate the calculated minimum (25th percentile −1.5 × IQR) and the calculated maximum (75th percentile +1.5 × IQR), and dots indicate the outliers beyond the calculated minima and maxima. Violins describe the kernel probability density of the underlying data. Significance (*P *< 0.05) is displayed as different letters and was determined by Tukey-adjusted estimated marginal means following a generalized additive mixed effects model (Fig. S8). Summary data are located in Excel Tables S21 and S22. GABA_B_R, GABA_B_ receptor; PFOS, perfluorooctanesulfonic acid; PAM, positive allosteric modulator; dpf, days post fertilization; D, dark; DMSO, dimethyl sulfoxide; IQR, interquartile range.

To examine whether PFOS acts as a GABAR antagonist, zebrafish larvae were exposed to one test concentration of the GABAR PAMs propofol (GABA_A_R) or CGP13501 (GABA_B_R), and 120 µM PFOS was applied 15 min later. An intermediate concentration of 1.38 µM CGP13501 was selected (Fig. 3H). Due to the strong hypoactivity phenotype induced by the lowest concentration of propofol tested (0.77 µM), as shown in Fig. 3C and D, one-quarter-log concentration lower (0.43 µM) was chosen for the co-exposure. Locomotor activity was assessed 60 min following PFOS exposure. In line with Figs. 1C and 2C, exposure to 120 µM PFOS resulted in dark-phase hyperactivity (Figs. 4B and D and 5B and D). Exposure to 0.43 µM propofol caused hypoactivity in the dark-phase (Fig. 4A and D). Co-exposure to 0.43 µM propofol and 120 µM PFOS blocked D1 and D2 PFOS-induced hyperactivity (Fig. 4C and D). The GABA_B_R PAM CGP13501 (1.38 µM) caused dark-phase hypoactivity (Fig. 5A and D) in line with Fig. 3G and H. PFOS-mediated hyperactivity was partially rescued in D1 and blunted to control levels in D2 by CGP13501 exposure (Fig. 5C and D).

### PFOS interferes with the function of GABARs in cultured mouse cortical neurons and human BrainSphere-derived networks

**Fig. 6.**
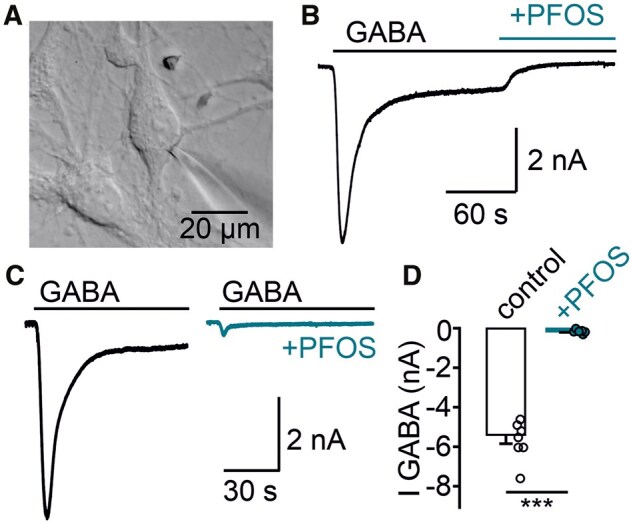
GABA_A_R-mediated currents are reduced in mouse cortical neurons exposed to PFOS. (A) Infrared differential interference contrast image of a patch-clamped cultured mouse cortical neuron. (B) Example experiment showing a representative pharmacologically isolated GABA_A_R-mediated response to 50 µM GABA that was inhibited by subsequent co-exposure of 120 µM PFOS. Extracellular solution was supplemented by 10 µM NBQX, 50 µM APV, and 3 µM CGP55845 to block AMPAR, NMDAR, and GABA_B_R, respectively. (C) Representative GABA currents in a control cell measured in control recording solution and a cell pre-treated and continuously superfused with PFOS containing solution. (D) Individual and mean GABA current (IGABA) in control cells (control, mean ± SE, *n* = 7) and cells pre-treated and during PFOS application (+PFOS, mean ± SE, *n* = 6). ***P = 0.001, Mann–Whitney *U*-test. Summary data are located in Excel Table S23. PFOS, perfluorooctanesulfonic acid; IGABA, GABA_A_ receptor-mediated current.

We aimed to provide multiple lines of evidence that PFOS antagonizes GABARs across model systems. To evaluate the antagonistic effect of PFOS on GABA_A_Rs in disassociated mouse cortical neurons, we measured pharmacologically isolated postsynaptic GABA_A_R-mediated chloride currents (Fig. 6A). In the control recording solution, application of 50 µM GABA induced a large postsynaptic chloride current (Fig. 6B to D). This GABAergic current was blocked by a subsequent wash-in of 120 µM PFOS, as shown for an example cell (Fig. 6B). In cells pre-treated and continuously superfused with PFOS-containing solution, GABA_A_R-mediated currents were substantially reduced compared with control cells, indicating an antagonistic effect of PFOS on GABA_A_R (Fig. 6C and D).

**Fig. 7.**
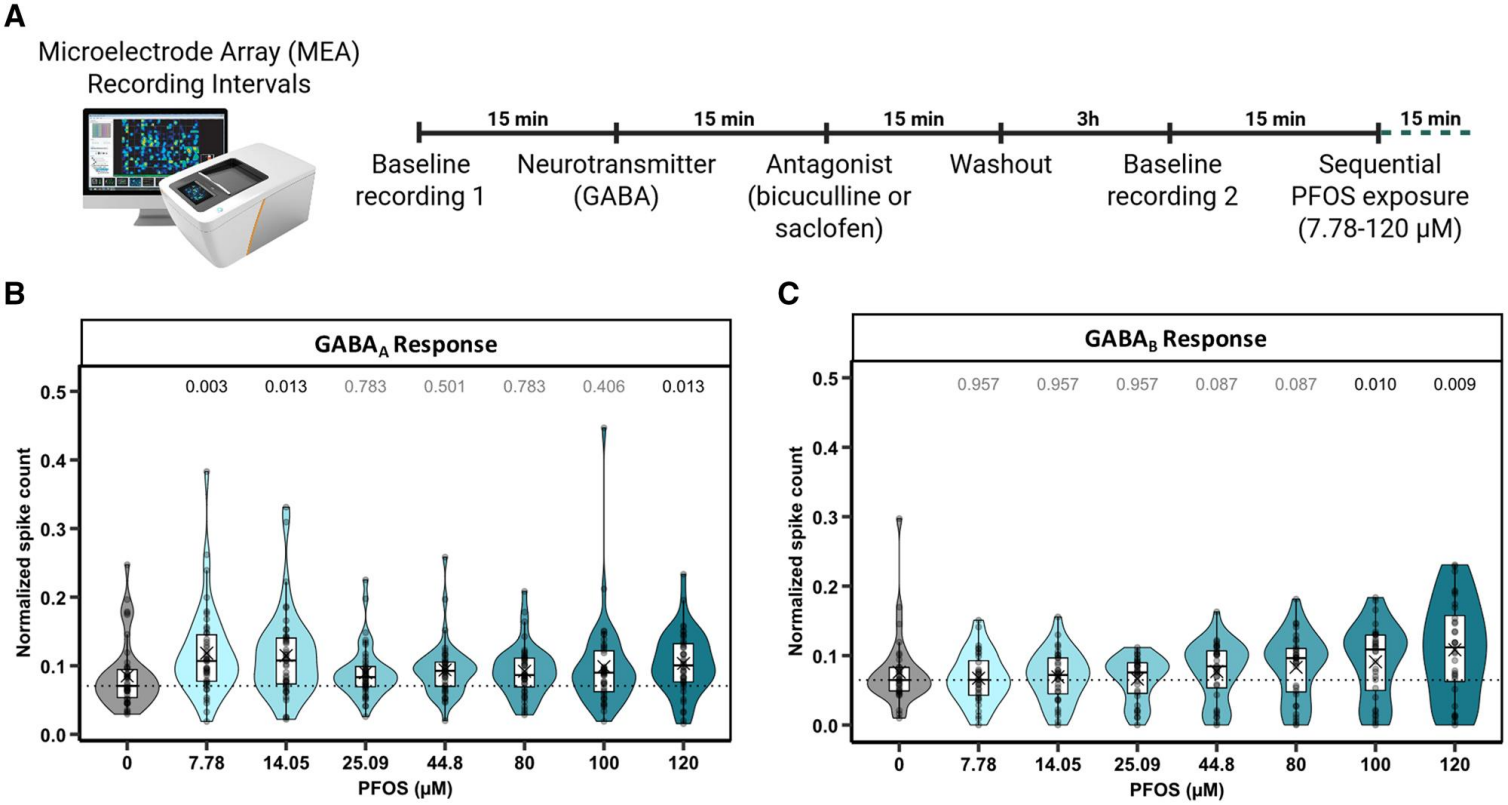
PFOS exposure increases spiking of GABA_A_R- and GABA_B_R-dependent units in human BrainSphere-derived neural networks. (A) Schematic representation of the exposure paradigm for GABA_A_ and GABA_B_ unit identification and PFOS treatment using microelectrode array (MEA) recordings. After baseline recording 1, the neurotransmitter GABA followed by the respective antagonist bicuculline or saclofen were applied for unit identification. Normalized spike count (*y*-axis) of BrainSpheres exposed to increasing concentrations of PFOS (*x*-axis) for (B) GABA_A_ and (C) GABA_B_ units. The dotted horizontal line represents the control median. Boxes indicate the median and interquartile range (IQR), X indicates the mean, whiskers indicate the calculated minimum (25th percentile −1.5 × IQR) and the calculated maximum (75th percentile +1.5 × IQR), and dots indicate the outliers beyond the calculated minima and maxima. Violins describe the kernel probability density of the underlying data. Values above violins represent adjusted *P*-values obtained from a Friedman test followed by a Conover post hoc test. *P*-values are Benjamini–Hochberg adjusted. Data inclusion criteria are visualized in the supplement (Fig. S9) (Owen 2025c). Summary data are located in Excel Table S24. PFOS, perfluorooctanesulfonic acid.

To extend the applicability of our findings to humans, we examined the effects of multiple concentrations of PFOS on GABA_A_R- and GABA_B_R-mediated responses in matured human BrainSphere-derived networks using the hMNR assay on MEAs. Following identification of GABA_A_R- and GABA_B_R-dependent units by response to the neurotransmitter GABA and respective antagonists bicuculline or saclofen, PFOS was gradually applied in increasing concentrations, and spontaneous network activity was recorded (Fig. 7A). Exposure to 7.78, 14.05, or 120 µM induced a significant increase in GABA_A_ spiking compared with the solvent control (Fig. 7B). PFOS-exposed GABA_B_ units exhibited a concentration-dependent increase in spiking relative to the solvent control with a significant effect observed at 100 and 120 µM PFOS (Fig. 7C). These data suggest that certain concentrations of PFOS are able to block the GABAR inhibitory current, thus leading to an excitation of the neural network in BrainSpheres.

**Fig. 8.**
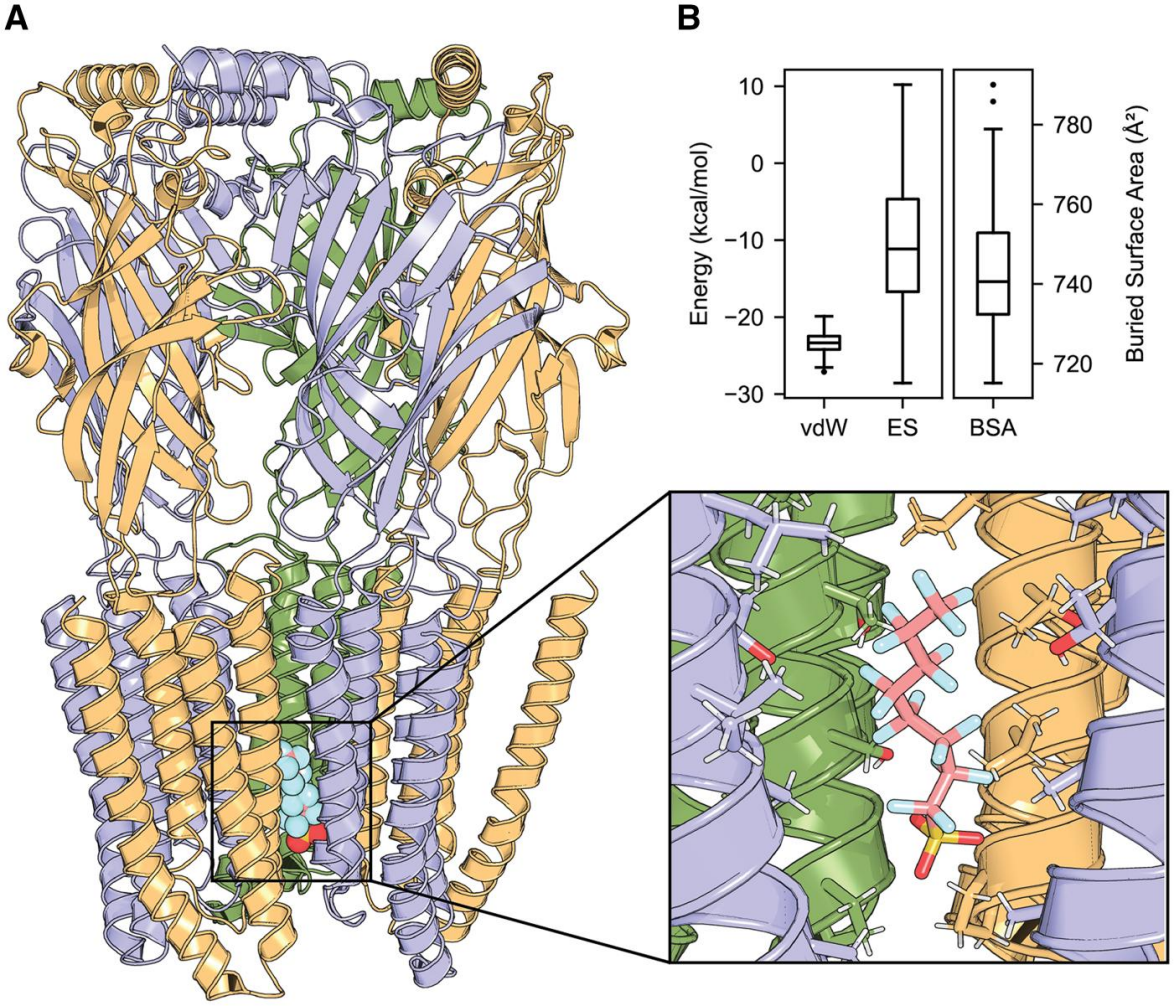
Possible binding of PFOS within the human α_1_β_2_γ_1_ GABA_A_R pore, as predicted by HADDOCK. The structure of GABA_A_R is based on PDB ID: 8SG0, modified to meet HADDOCK input requirements. (A) Cartoon representation of the highest ranked structure within the best-ranked cluster with α-subunits in yellow, β-subunits in blue, and the γ-subunit in green. The inset displays PFOS as stick representation in its predicted binding position. (B) Energy analysis of the 91 structures within the best ranked cluster. The binding interface of PFOS within the GABA_A_R pore has an extensive buried surface area (BSA) and is mainly stabilized by van der Waals (vdW) forces, whereas electrostatics (ES) have a minor contribution. Boxes indicate the median and interquartile range (IQR), whiskers indicate the calculated minimum (25th percentile −1.5 × IQR) and the calculated maximum (75th percentile +1.5 × IQR), and dots indicate the outliers beyond the calculated minima and maxima. PFOS, perfluorooctanesulfonic acid; vdW, van der Waals; ES, electrostatics; BSA, buried surface area.

Based on electrophysiological recordings in *Xenopus* oocytes, Tukker et al. (2020) hypothesized that PFOS may bind within the channel pore of the GABA_A_R, thereby blocking the flow of chloride ions and producing an antagonistic effect. To preliminarily determine whether this interaction is possible, we performed molecular docking of PFOS to the human α_1_β_2_γ_1_ GABA_A_R pore using the HADDOCK 2.4 web server (Honorato et al. 2021, 2024). HADDOCK clustered 197 out of 200 structures into two clusters, representing 98% of the water-refined models generated. The highest ranked structure within the best-ranked cluster shows that PFOS blocks the channel pore with its sulfonate group directed toward the intracellular opening of the pore (Fig. 8A). Energy analysis of the best ranked cluster reveals that the binding interface of PFOS within the GABA_A_R pore has a large buried surface area (BSA) and is mainly stabilized by van der Waals forces with a minor contribution from electrostatic interactions (Fig. 8B). This provides initial insight into a potential mechanism of PFOS inhibition of the most common human GABA_A_R subunit combination.

## Discussion

**Fig. 9.**
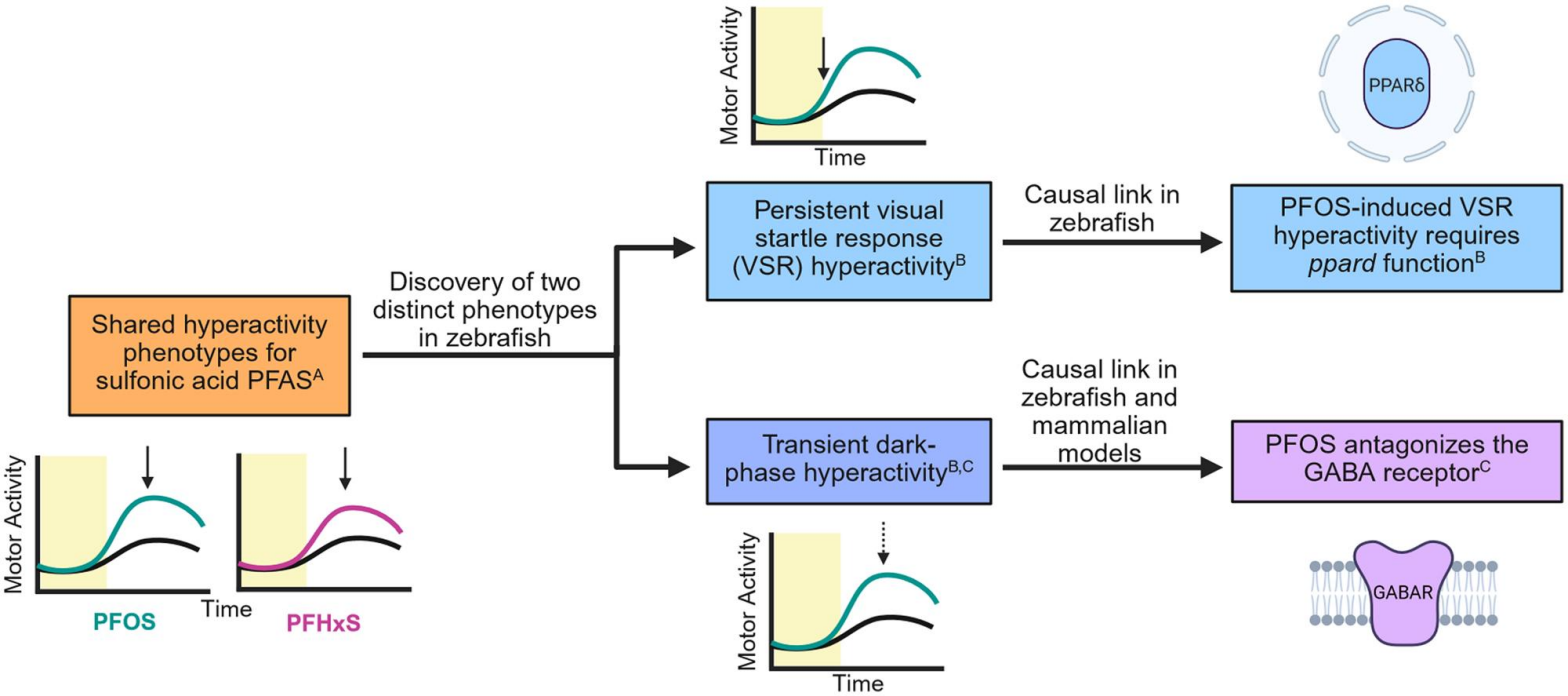
Discovery of mechanisms underlying sulfonic acid PFAS-induced behavioral phenotypes. This study aimed to build on previous work revealing a shared hyperactivity phenotype for sulfonic acid PFAS^A^ (Gaballah et al. 2020). Visual startle response (VSR) hyperactivity was found to persist to 8 dpf and require *ppard* function^B^ (Gutsfeld et al. 2024). Transient dark-phase hyperactivity was hypothesized to be mediated by a chemical-receptor interaction (Gutsfeld et al. 2024), leading to the examination of PFOS antagonism of the GABARs in larval zebrafish and mammalian models in the current study^C^ (Owen 2025b). PFAS, per- and polyfluoroalkyl substances; PFOS, perfluorooctanesulfonic acid; PFHxS, perfluorohexanesulfonic acid; VSR, visual startle response; dpf, days post fertilization.

We examined the potential for acute PFOS exposure to cause rapid dark-phase hyperactivity through interactions with GABARs across vertebrate systems, building on previous findings in larval zebrafish (Fig. 9). The assessment of larval zebrafish locomotor activity is commonly used to rapidly investigate the effects of chemicals on neurobehavior, as larvae exhibit stereotyped behaviors in response to stimuli, which may change with chemical exposure. Alterations in behavior can therefore indicate disruption in the development or function of underlying circuits that control these stereotyped behaviors. The light–dark transition test has previously captured behavioral alterations following acute and developmental exposure to PFAS, including shared phenotypes in a subset of compounds containing the sulfonic acid moiety (Gaballah et al. 2020; Menger et al. 2020; Rericha et al. 2021; Wu et al. 2022; Gutsfeld et al. 2024). Exposure to PFOS or PFHxS was shown to cause dark-phase and visual startle response hyperactivity under the same exposure paradigms and similar global changes in transcriptomics, indicating potential shared mechanisms of action for the perfluoroalkyl sulfonates (Gutsfeld et al. 2024). In this study, we replicated previously reported acute dark-phase hyperactivity in PFOS-exposed larvae, determined the time-of-peak-effect of acute PFOS exposure, and showed that dark-phase hyperactivity is transient. These data support the concept that PFOS interacts with receptor-dependent signaling to transiently provoke increased locomotor activity during the dark period.

In this study, we demonstrated that GABAR modulation influences dark-phase swimming behavior in 5 dpf larval zebrafish, providing insight into a mechanism underlying the stereotypic response of heightened motor activity in the dark following a light period. The ionotropic GABA_A_R is a chloride channel and the primary inhibitory receptor of the central nervous system. GABA_A_R structure and function are conserved among vertebrates, with 17/19 mammalian receptor subunits sharing one or more orthologs with the 23 identified subunits in zebrafish (Cocco et al. 2017; Monesson-Olson et al. 2018; Sadamitsu et al. 2021). In zebrafish, loss-of-function mutations in GABA_A_R α or γ_2_ subunits induced hyperactivity at 2 (Barnaby et al. 2022) or 5 (Liao et al. 2019) dpf, respectively, suggesting proper GABA_A_R signaling is required for locomotion in early life-stage zebrafish. The application of GABA_A_R antagonists such as pentylenetetrazole (PTZ) in wild-type zebrafish ≤7 dpf was reported to chemically induce hyperactivity and convulsions, which could be reversed by co-treatment with PAMs of the GABA_A_R (Baraban et al. 2005; Baxendale et al. 2012; Bandara et al. 2020). In line with these findings, we showed that the GABA_A_R antagonist picrotoxin and GABA_A_R PAM propofol have opposing influences on dark-phase locomotor activity. Picrotoxin phenocopied the behavioral profile of PFOS, providing initial evidence that PFOS may act as a GABA_A_R antagonist to produce the observed behavioral changes. To causally demonstrate PFOS-induced hyperactivity is dependent on the inhibition of the GABA_A_R, we pre-treated PFOS-exposed larvae with propofol and reported a full reversal of the dark-phase hyperactivity phenotype. Taken together, these results support a mechanism–behavior relationship between PFOS antagonism of the GABA_A_R and dark-phase hyperactivity in larval zebrafish for the first time.

We expanded our investigation of the interaction between PFOS and the GABARs by assessing its human relevance through electrophysiology approaches in mammalian models. In cultured mouse cortical neurons, acute PFOS exposure significantly reduced GABA_A_R-mediated current, suggesting an antagonistic effect of PFOS on the rodent GABA_A_R. This effect is unlikely to be attributed to cytotoxicity, as the reduction in GABA_A_R-induced current followed an immediate wash-in of PFOS in one example recording. We further demonstrated that acute PFOS exposure increases spiking of GABA_A_R- and GABA_B_R-dependent units in an hiPSC-based model, indicating an antagonistic effect of PFOS on both GABAR subtypes. Preliminary docking of PFOS to the human α_1_β_2_γ_1_ GABA_A_R channel pore provides insight into one potential mechanism of PFOS inhibition of the GABA_A_R. In support of our findings, one study revealed that PFOS exposure (0.1 to 100 µM) in *Xenopus* oocytes expressing the human α_1_β_2_γ_2L_ GABA_A_R inhibited GABA-evoked ion currents (LOEC; 0.1 µM) (Tukker et al. 2020). PFOS (100 µM) also induced the hyperexcitation of rat primary cortical neurons, although the opposite effect was observed in hiPSC-derived neuron cultures (Tukker et al. 2020). This contradictory finding may be due to the differing composition of neuronal subtypes in the hiPSC cultures compared with the BrainSphere-derived networks, as the latter has a more diverse range of neurotransmitters present in the network and a bias toward inhibitory GABAergic neurons. Collectively, we provide evidence that PFOS interacts with the GABA_A_R and GABA_B_R at the molecular level in mammalian models. In rodents, lactational exposure to PFOS triggered increased GABA and glutamate levels in the hippocampus and reduced learning in male mice (Mshaty et al. 2020). Another study determined that GABA and glycine levels were elevated in the cortex of Sprague-Dawley rats prenatally exposed to PFOS, which was linked to hyperactivity and increased thigmotaxis (Reardon et al. 2019). This suggests that PFOS interaction with GABARs further results in functional consequences in mammalian systems.

In rodents, genetic ablation and pharmacological manipulation of the metabotropic GABA_B_R induces behavioral changes and seizures (Joshi et al. 2016). The role of the GABA_B_R in larval zebrafish locomotion has not been extensively characterized, although both GABA receptor subtypes are present in larvae and adults (Cocco et al. 2017; Song et al. 2017). Adult zebrafish injected with 0.5 mg/kg of the GABA_B_R antagonist saclofen exhibited no behavioral alterations compared with the control (Assad et al. 2020). Similarly, we did not observe any effects on locomotor activity with exposure to saclofen, which may be attributed to a lack of neuroactivity at the tested concentrations in this model organism. We did, however, show that the GABA_B_R is involved in the control of locomotion in the zebrafish by linking exposure to the GABA_B_R PAM CGP13501 with dark-phase hypoactivity. Moreover, co-exposure to CGP13501 and PFOS partially rescued the D1 hyperactivity phenotype induced by PFOS and fully reversed it in D2, highlighting an interaction between PFOS and the GABA_B_R. To our knowledge, there are currently no other reports indicating the involvement of the GABA_B_R in PFOS-mediated neuroactivity in zebrafish. Our results suggest that this GABAR subtype should be considered as a potential mediator of neurobehavioral outcomes in zebrafish.

In addition to PFOS antagonism of GABARs, PFOS likely has other neurological targets that interplay with GABAR signaling. Crosstalk between GABARs, glutamate receptors (Potapenko et al. 2013; Wen et al. 2022), and dopamine receptors (Flores-Hernandez et al. 2000; Seamans et al. 2001) may mediate the behavioral phenotypes observed in PFOS-exposed larvae. PFOS disrupts the function and expression of glutamate receptor subtypes NMDARs and AMPARs in rat hippocampal (Liao et al. 2009; Wang et al. 2019; Zhang et al. 2019), cortical (Ishida et al. 2017), and cerebellar (Berntsen et al. 2018) neurons, producing a calcium influx that could lead to excitotoxicity. PFOS exposure was also reported to increase intracellular calcium concentrations by inositol 1,4,5-triphosphate receptor (Liu et al. 2011; Yang et al. 2024) and L-type calcium channel (Liao et al. 2008) activation. A study conducted in 4 dpf zebrafish suggested that PFOS-induced behavioral hyperactivity may be linked, in part, to the activation of ryanodine receptors and release of intracellular calcium stores (Christou et al. 2020). PFOS additionally targets the dopaminergic system by altering dopamine levels and dopamine receptor expression in rodents (Salgado et al. 2016), northern leopard frogs (Foguth et al. 2019), and east Greenland polar bears (Eggers Pedersen et al. 2015). Lower concentrations of PFOS were required to induce robust dopaminergic neurodegeneration than for GABAergic, serotoninergic, or cholinergic neurons in *Caenorhabditis elegans*, suggesting high sensitivity of the dopaminergic system to PFOS, potentially via mitochondrial dysfunction (Sammi et al. 2019). PFOS-exposed larval zebrafish displayed abnormal behavior, altered dopamine-related gene expression (Wu et al. 2022; Mahapatra et al. 2023), and subpallial dopaminergic neuronal loss (Kalyn et al. 2023). These PFOS-induced alterations to the glutamatergic and dopaminergic systems may produce downstream effects that impact GABAergic action. Here, however, we show a direct mechanistic link between PFOS and GABARs, which suggests that PFOS binding GABARs constitutes one molecular initiating event for adverse neurological outcomes in PFOS-exposed organisms.

In comparison with a 2016 to 2017 NHANES (CDC 2023) and a 2016 Health Outcomes and Measures of the Environment (HOME) study (Vuong et al. 2016), the concentration of PFOS used here exceeds levels found in human blood by approximately 3 to 4 orders of magnitude. Developmental exposure from 1 to 4 dpf provoked dark-phase hyperactivity at a lower nominal concentration of PFOS (2.47 µM) (Gutsfeld et al. 2024), likely due to bioaccumulation of the chemical increasing its toxic potential (Vogs et al. 2019; Menger et al. 2020; Tal and Vogs 2021). Acute exposures at 5 dpf, therefore, require the application of a higher concentration to observe the same effect. Along with the exposure window, it is equally important to consider that humans are chronically exposed to chemicals in complex mixtures, in which effects on nervous system function may occur at concentrations less than the effect threshold for individual chemicals (Braun et al. 2024). Biomonitoring data consistently shows that multiple classes of PFAS are detected in human blood with PFOS dominating relative to other identified PFAS (Yeung et al. 2008; Poothong et al. 2017; Göckener et al. 2020). Mixture studies in animal models and cell lines have demonstrated the potential of different PFAS components to act synergistically, antagonistically, or additively based on the ratios and number of chemicals present (Kjeldsen and Bonefeld-Jørgensen 2013; Hu et al. 2014; Hoover et al. 2019; Ojo et al. 2020; Ojo et al. 2021; Pierozan et al. 2023; Ríos-Bonilla et al. 2024). By investigating the underlying mechanisms of individual PFAS, our confidence in predicting mixture toxicity for human health risk assessment can be strengthened.

Multiple regulatory agencies develop Adverse Outcome Pathways (AOPs) to build mode of action frameworks for chemical risk assessment based on toxicological data (Ankley et al. 2010; OECD 2018). Given enough underlying evidence, AOPs can be implemented in an Integrated Approach to Testing and Assessment (Tollefsen et al. 2014). The objective is to causally link chemical exposure with key biological events and adverse outcomes (Collier et al. 2016). In this study, we used pharmacological manipulation and electrophysiology recordings to causally demonstrate that GABAR antagonism is a conserved molecular initiating event across taxa exposed to PFOS. By demonstrating the translational relevance of zebrafish for evaluating chemical impacts on the nervous system, we build confidence in the use of the 3R (Hubrecht and Carter 2019)-compliant early life-stage zebrafish model for the detection of human-relevant neurotoxicants. More broadly, the strategy used here can reduce uncertainty in the use of mode of action frameworks in regulatory contexts.

## Supplementary Material

kfaf101_Supplementary_Data

## Data Availability

Data are available at https://doi.org/10.5281/zenodo.15394335 (Owen et al. 2025).
