# Supplementary material for: Perfluorooctanesulfonic acid (PFOS) antagonizes gamma-aminobutyric acid (GABA) receptors in larval zebrafish and mammalian models
Source: Toxicol Sci. 2025 Jul 23;207(2):449–66. doi: 10.1093/toxsci/kfaf101 (PMC12560803; doi:10.1093/toxsci/kfaf101)
Supplement: kfaf101_Supplementary_Data [file kfaf101_supplementary_data.zip › kfaf101_Supplementary_Data/toxsci-25-0273-File011.pdf]

Supplemental Material

Perfluorooctanesulfonic acid (PFOS) antagonizes gamma-aminobutyric acid (GABA) receptors in larval zebrafish and mammalian models.

Renee Owen[1], Gabriel de Macedo[2*], Jana Nerlich[3*], Ilka Scharkin[4*], Kristina Bartmann[4,5], Jonas Döbler[6], Beatrice Engelmann[7], Ulrike E. Rolle-Kampczyk[7], David Leuthold[1], Sebastian Gutsfeld[1], Nicole Schweiger[1], Tamara Tal[1,8#]

[1]Department of Ecotoxicology, Chemicals in the Environment Research Section, Helmholtz-Centre for Environmental Research – UFZ, Leipzig, Germany

[2]Department of Molecular Biology and Biochemistry, Federal University of Santa Maria, Santa Maria, Brazil

[3]Medical Faculty, Carl Ludwig Institute of Physiology, University of Leipzig, Leipzig, Germany

[4]IUF – Leibniz Research Institute for Environmental Medicine, Düsseldorf, Germany

[5]DNTOX GmbH, Düsseldorf, Germany

[6]Institute of Biochemistry and Biotechnology, Martin Luther University Halle-Wittenberg, Halle/Saale, Germany

[7]Department of Molecular Toxicology, Chemicals in the Environment Research Section, Helmholtz-Centre for Environmental Research – UFZ, Leipzig, Germany

[8]Medical Faculty, University Leipzig, Leipzig, Germany

[#]Corresponding Author: Helmholtz Center for Environmental Research – UFZ, Permoserstrasse 15, 04318 Leipzig, Germany. Email: tamara.tal@ufz.de

*Equal contribution

Supplemental Figures

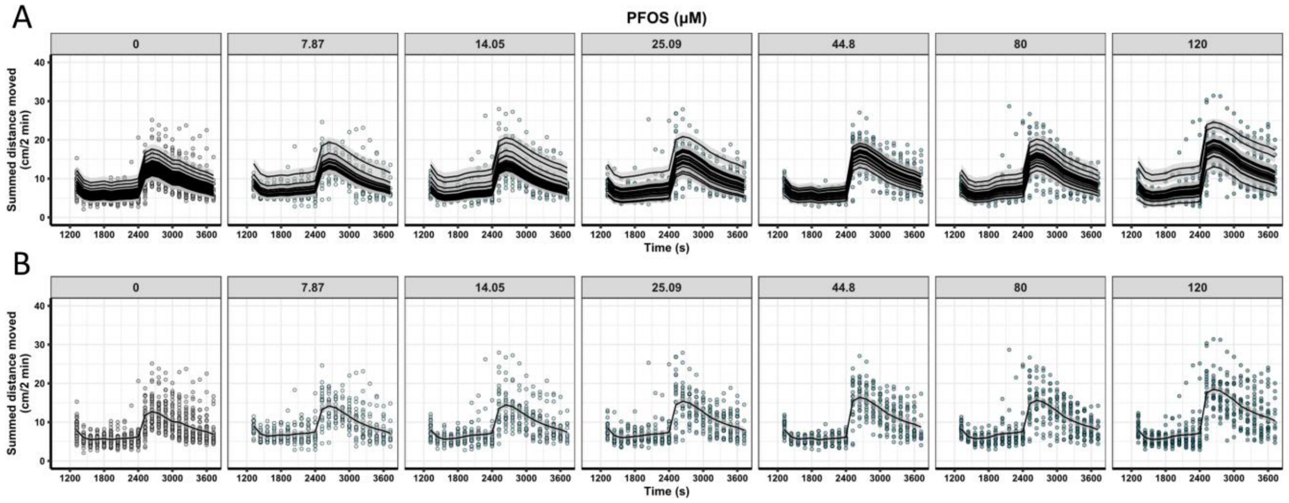

Figure S1: Generalized Additive Mixed Effects Model (GAMM) Visualization for Figure 1C. (A) visualizing random effects, where each line represents one larva, or **(B)** without visualizing random effects. Grey area around lines represent 95% confidence intervals. Summary data can be found in supplemental Excel Table S1.

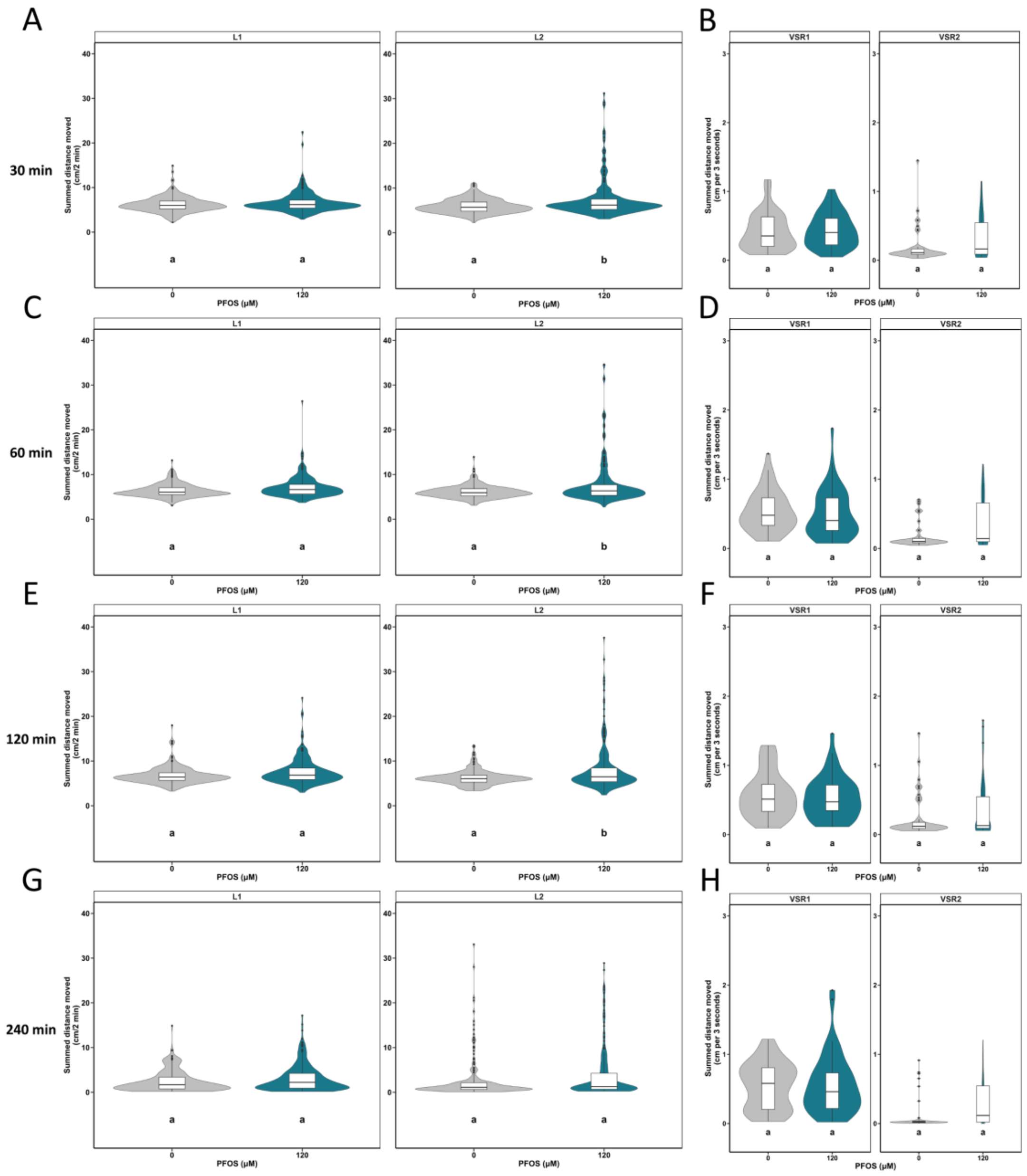

Figure S2: L1 and L2 phase motor activity and Visual Startle Response (VSR) activity in 5 dpf PFOS-exposed larvae corresponding to Figure 2A-D. (A,C,E,G) Box- and violin-plots signifying the distance moved (cm) in 2-min periods across the first 10 min in the light (L1) and the next 10 min in the light (L2) at 13,238 lux for each larva exposed to 120 µM PFOS (blue) or 0.4% DMSO (grey) for **(A)** 30 min, **(C)** 60 min, **(E)**, 120 min, or **(G)** 240 min before locomotor assessment. **(B,D,F,H)** Box- and violin plots of motor activity (cm) in the 3 s following the dark-light (VSR1) or light-dark (VSR2) transition following 120 µM PFOS exposure or 0.4% DMSO. Replicate numbers range from 45-47 larvae per test group. Boxes indicate the median and IQR, whiskers indicate the calculated minimum (25th percentile -1.5 x IQR) and the calculated maximum (75th percentile +1.5 x IQR), and dots indicate the outliers beyond the calculated minima and maxima. Violins describe the kernel probability density of the underlying data. Significance ($p < 0.05$) is displayed as different letters and was determined by Tukey-adjusted estimated marginal means following a generalized additive mixed effects model. Summary data is located in supplemental Excel Tables S4-S13. Note: L, light; VSR, visual startle response; DMSO, dimethyl sulfoxide; PFOS, perfluorooctanesulfonic acid; IQR, interquartile range.

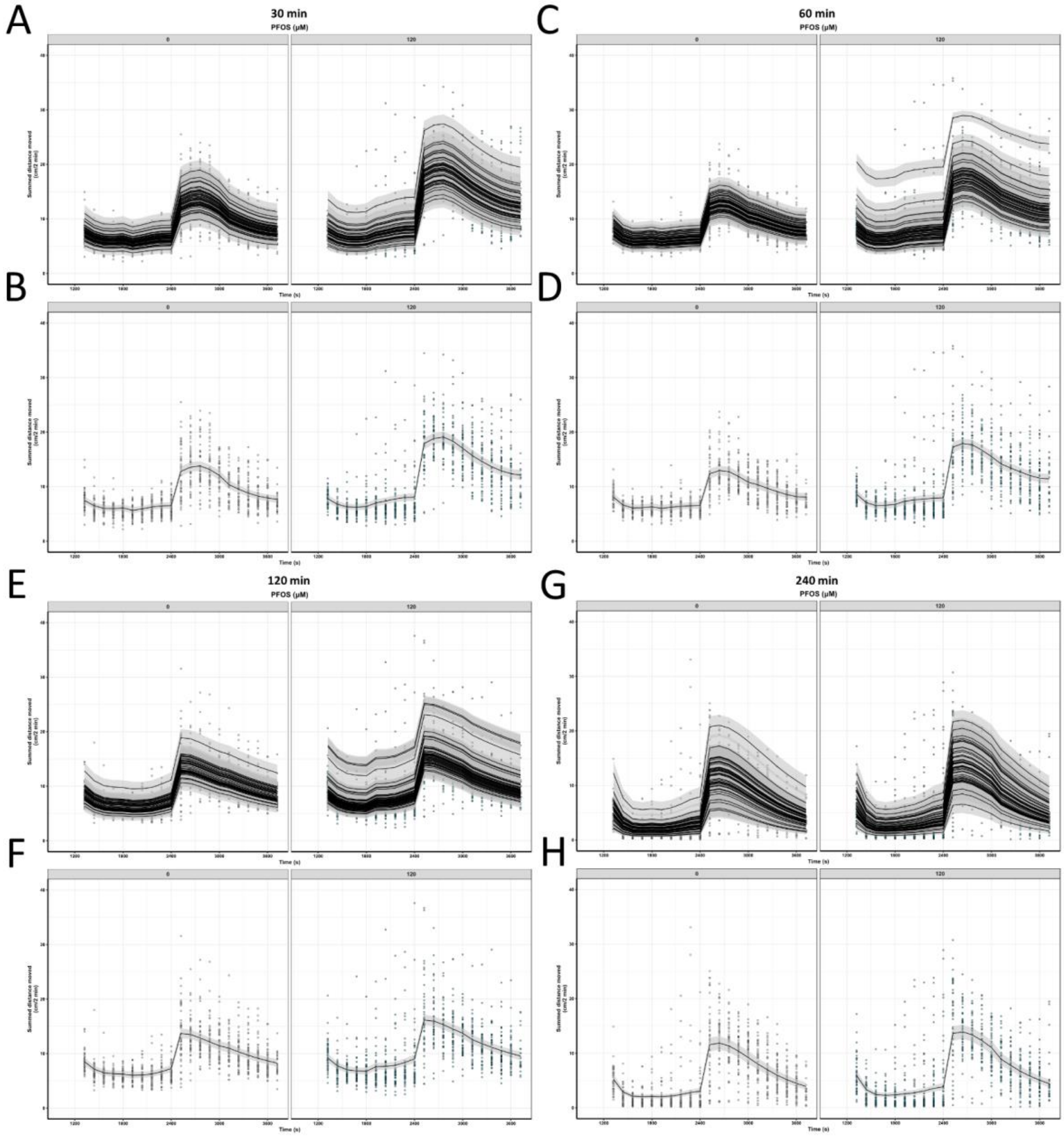

Figure S3: GAMM Visualization for Figure 2A-D and Figure S2A,C,E,G. (A,C,E,G) visualizing random effects, where each line represents one larva, or **(B,D,F,H)** without visualizing random effects. Grey area around lines represent 95% confidence intervals. Summary data can be found in supplemental Excel Tables S4-S7.

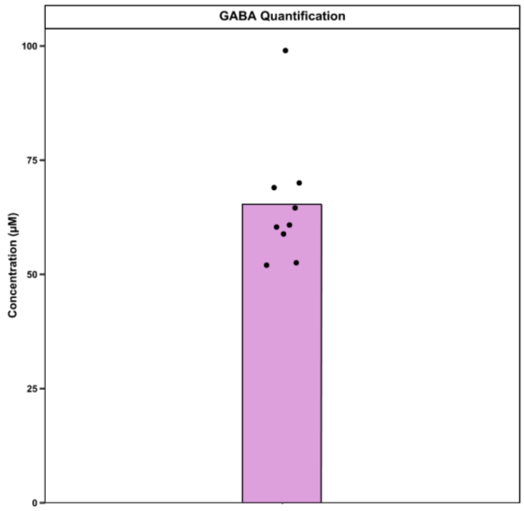

Figure S4: GABA levels in 5 dpf larval zebrafish tissue. Bar plot of mean GABA concentration (µM) detected in pooled (n = 40) untreated larval zebrafish of the TL strain via mass spectrometry and multiple reaction monitoring (MRM) (9 replicates). Each dot represents a biological replicate.

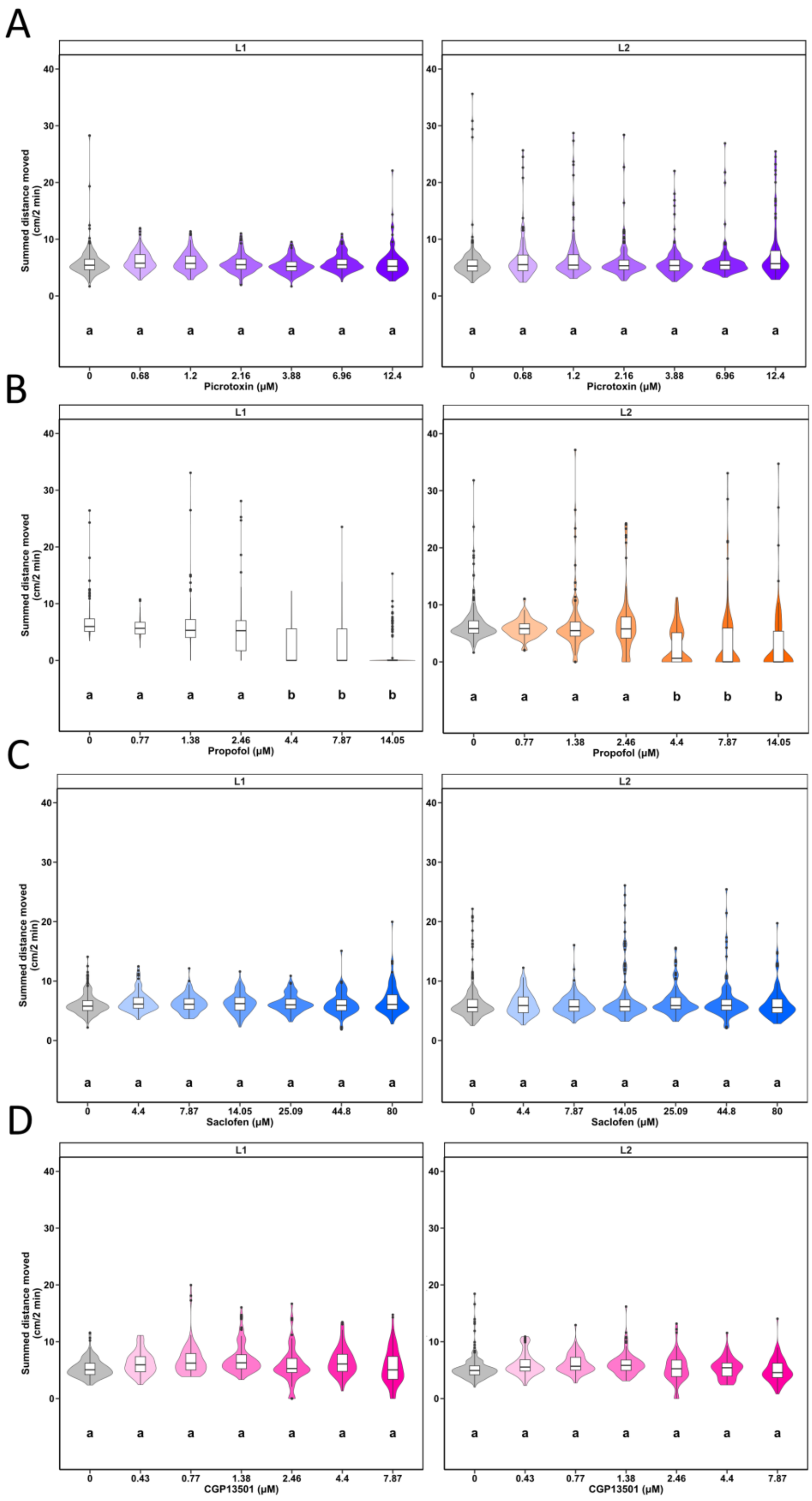

Figure S5: L1 and L2 phase motor activity in 5 dpf larvae corresponding to Figure 3A-H. Box- and violin-plots signifying the distance moved (cm) in 2-min periods across the first 10 min in the light (L1) and the next 10 min in the light (L2) at 13,238 lux for each larva exposed to **(A)** 0.88-12.4 µM picrotoxin, **(B)** 0.77-14.05 µM propofol, **(C)** 4.4-80 µM saclofen, or **(D)** 0.43-7.87 µM CGP13501 or 0.4% DMSO (grey). Replicate numbers range from 13-48 larvae per test group. Boxes indicate the median and IQR, whiskers indicate the

calculated minimum (25th percentile -1.5 x IQR) and the calculated maximum (75th percentile +1.5 x IQR), and dots indicate the outliers beyond the calculated minima and maxima. Violins describe the kernel probability density of the underlying data. Significance ($p < 0.05$) is displayed as different letters and was determined by Tukey-adjusted estimated marginal means following a generalized additive mixed effects model. Summary data is located in supplemental Excel Tables S14-S18. Note: L, light; DMSO, dimethyl sulfoxide; PFOS, perfluorooctanesulfonic acid; IQR, interquartile range.

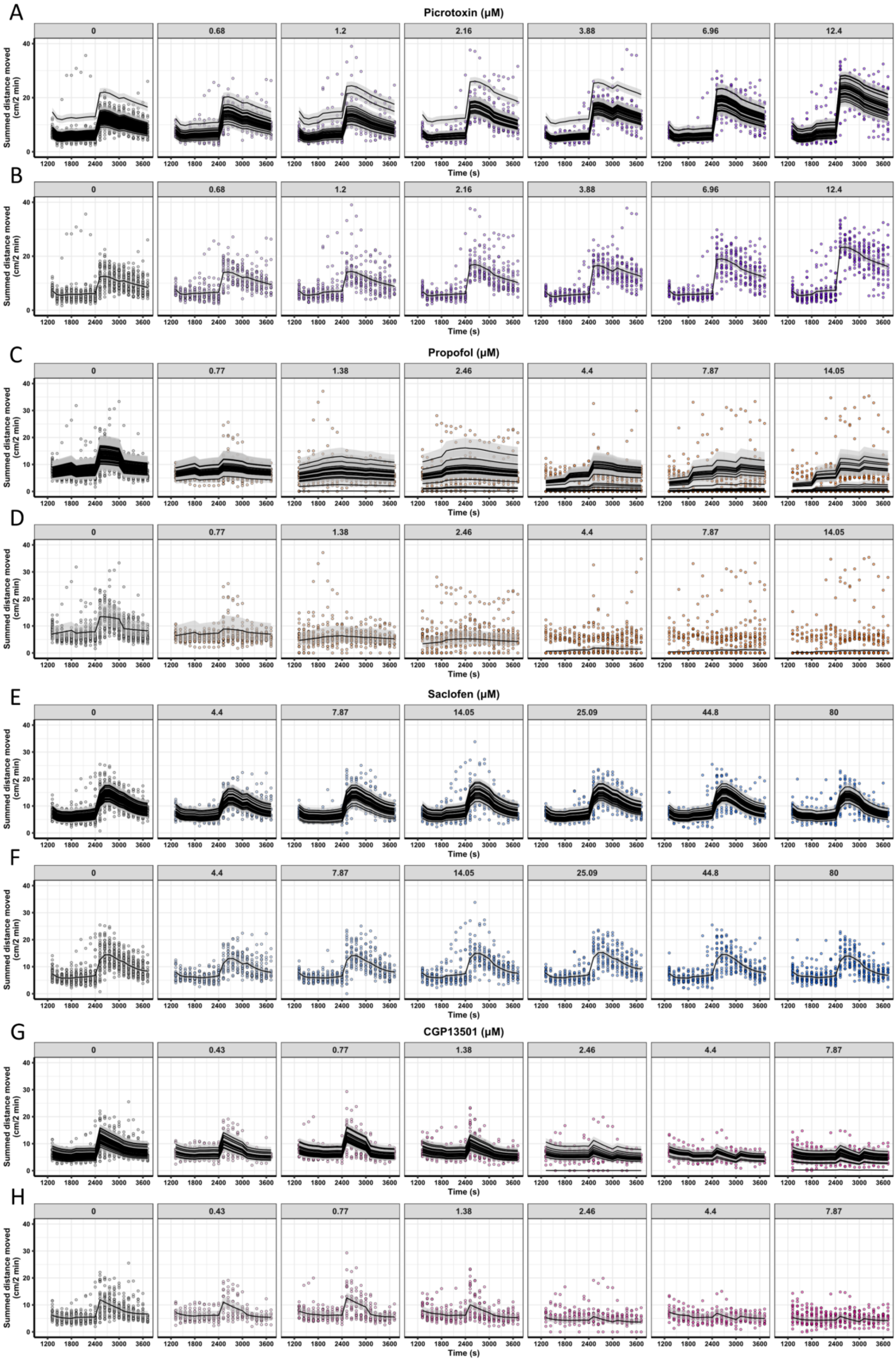
A
B
C
D
E
F
G
H
Picrotoxin (µM)
Propofol (µM)
Saclofen (µM)
CGP13501 (µM)
Summed distance moved (cm/2 min)
Time (s)

Figure S6: GAMM Visualization for Figure 3A-H and Figure S5A-D. (A,C,E,G) visualizing random effects, where each line represents one larva, or **(B,D,F,H)** without visualizing random effects. Grey area around lines represent 95% confidence intervals. Summary data can be found in supplemental Excel Tables S14-S18.

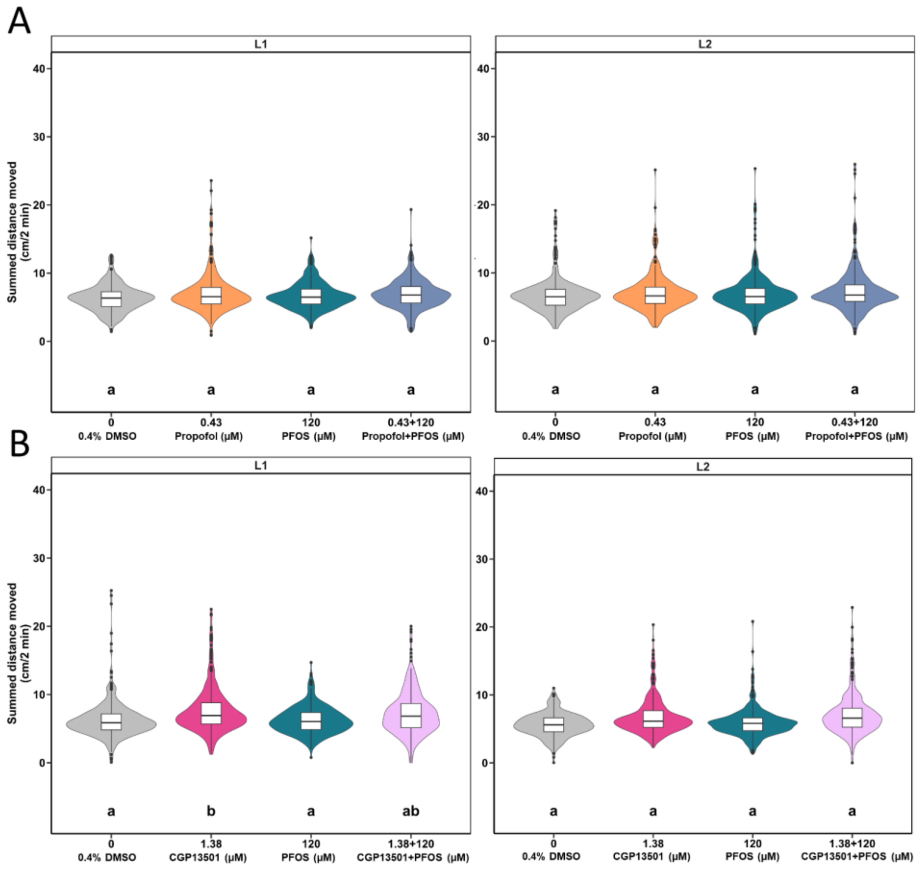

Figure S7: L1 and L2 phase motor activity in 5 dpf larvae corresponding to Figure 4A-D and Figure 5A-D. Box- and violin-plots signifying the distance moved (cm) in 2-min periods across the first 10 min in the light (L1) and the next 10 min in the light (L2) at 13,238 lux for each larva. **(A)** Larvae exposed to 0.43 µM GABAAR PAM propofol (orange; n = 68), 120 µM PFOS (blue; n = 72), or propofol and PFOS (lavender-blue; n = 69) compared to 0.4% DMSO control (grey; n = 67). **(B)** Larvae exposed to 1.38 µM GABABR PAM CGP13501 (pink; n = 65), 120 µM PFOS (blue; n = 67), or the co-exposure of CGP13501 and PFOS (lilac; n = 67) compared to 0.4% DMSO control (grey; n = 70). Boxes indicate the median and IQR, whiskers indicate the calculated minimum (25th percentile - 1.5 x IQR) and the calculated maximum (75th percentile +1.5 x IQR), and dots indicate the outliers beyond the calculated minima and maxima. Violins describe the kernel probability density of the underlying data. Significance ($p < 0.05$) is displayed as different letters and was determined by Tukey-adjusted estimated marginal means following a generalized additive mixed effects model. Summary data is located in supplemental Excel Tables S19-S22. Note: L, light; DMSO, dimethyl sulfoxide; PFOS, perfluorooctanesulfonic acid; IQR, interquartile range.

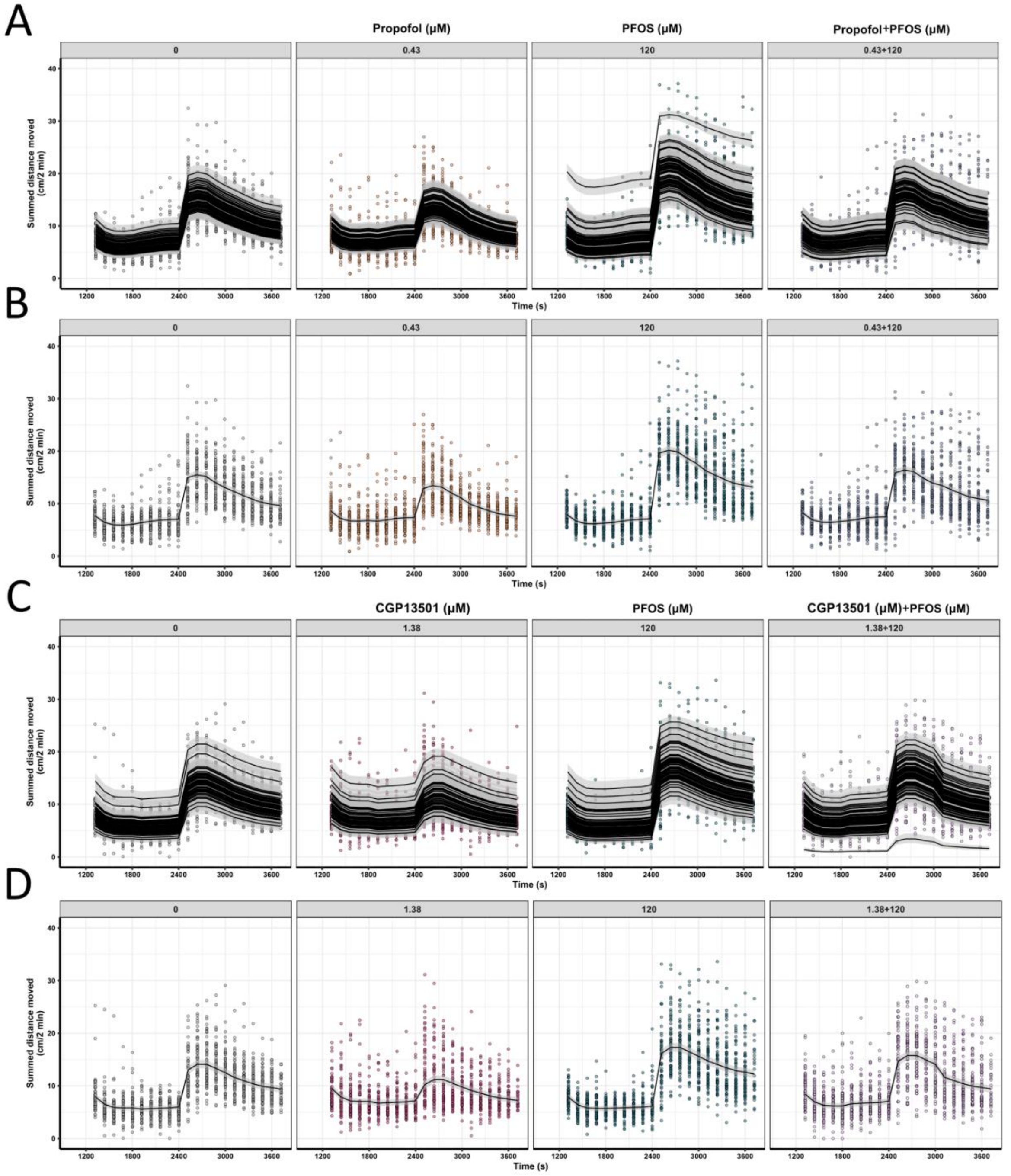

Figure S8: GAMM Visualization for Figure 4D, 5D, S7A,B. (A,C) visualizing random effects, where each line represents one larva, or **(B,D)** without visualizing random effects. Grey area around lines represent 95% confidence intervals. Summary data can be found in supplemental Excel Tables S19-S22.

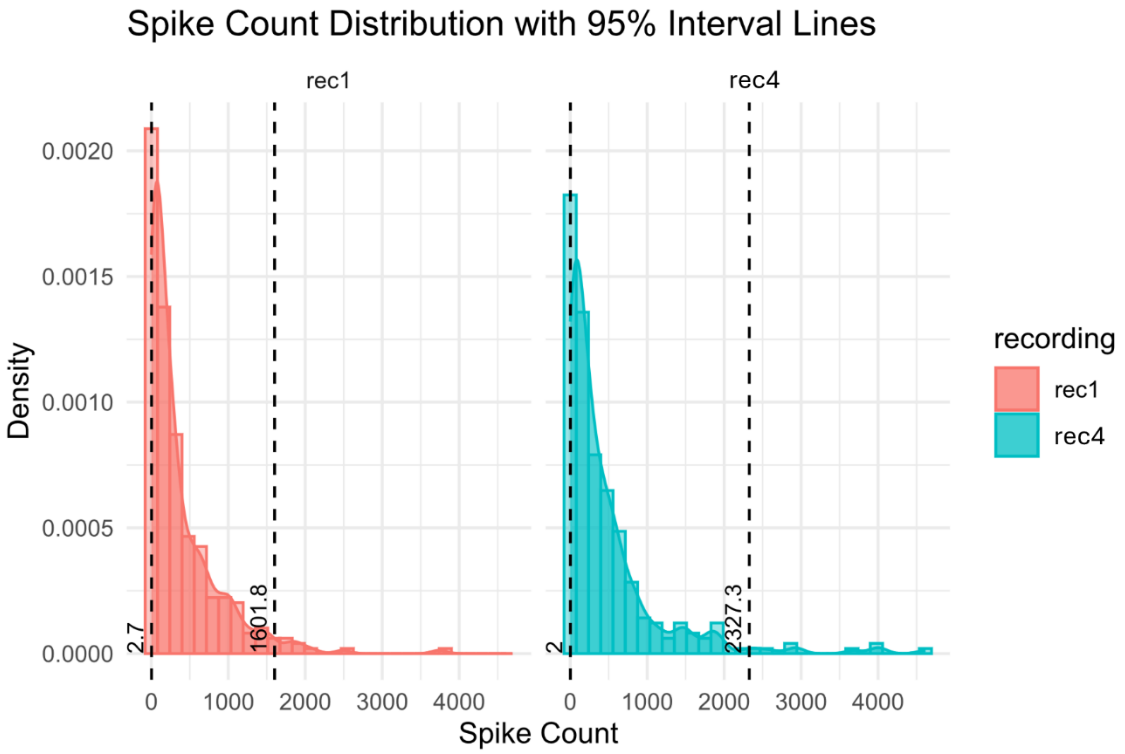

Figure S9: Spike count distributions with 95% confidence intervals for baseline recordings 1 and 4 corresponding to Figure 7B,C. For each baseline recording, the 2.5th and 97.5th percentiles (dashed vertical lines) were calculated to provide a non-parametric estimate of the 95% confidence interval bounds. Prior to quantile estimation, recordings were filtered to exclude non-firing or inactive units; specifically, only entries with spike counts greater than zero in both the initial baseline (recording 1) and the second baseline (recording 4) were retained. Only recordings with spike counts between ≥3 and ≤1601 per 15 minutes in the initial baseline (recording 1), and between ≥2 and ≤2327 in the second baseline (recording 4), were included in the analysis (n = 310).
